# THP-1 macrophage cholesterol efflux is impaired by palmitoleate through Akt activation

**DOI:** 10.1371/journal.pone.0233180

**Published:** 2020-05-21

**Authors:** Jenika D. Marshall, Emily R. Courage, Ryan F. Elliott, Madeline N. Fitzpatrick, Anne D. Kim, Andrea F. Lopez-Clavijo, Bronwyn A. Woolfrey, Mireille Ouimet, Michael J. O. Wakelam, Robert J. Brown

**Affiliations:** 1 Department of Biochemistry, Memorial University of Newfoundland, Newfoundland and Labrador, Canada; 2 University of Ottawa Heart Institute, Ottawa, Ontario, Canada; 3 The Babraham Institute, Cambridge, England, United Kingdom; 4 Department of Biochemistry, Microbiology and Immunology, University of Ottawa, Ottawa, Ontario, Canada; University of Milano, ITALY

## Abstract

Lipoprotein lipase (LPL) is upregulated in atherosclerotic lesions and it may promote the progression of atherosclerosis, but the mechanisms behind this process are not completely understood. We previously showed that the phosphorylation of Akt within THP-1 macrophages is increased in response to the lipid hydrolysis products generated by LPL from total lipoproteins. Notably, the free fatty acid (FFA) component was responsible for this effect. In the present study, we aimed to reveal more detail as to how the FFA component may affect Akt signalling. We show that the phosphorylation of Akt within THP-1 macrophages increases with total FFA concentration and that phosphorylation is elevated up to 18 hours. We further show that specifically the palmitoleate component of the total FFA affects Akt phosphorylation. This is tied with changes to the levels of select molecular species of phosphoinositides. We further show that the total FFA component, and specifically palmitoleate, reduces apolipoprotein A-I-mediated cholesterol efflux, and that the reduction can be reversed in the presence of the Akt inhibitor MK-2206. Overall, our data support a negative role for the FFA component of lipoprotein hydrolysis products generated by LPL, by impairing macrophage cholesterol efflux via Akt activation.

## Introduction

Lipoprotein lipase (LPL) is an extracellular enzyme required for the hydrolysis of triacylglycerols (TAG), and to a much lesser extent phospholipids, from TAG-rich lipoproteins within the bloodstream [1,2]. LPL is anchored to the outside of the endothelial cells lining the luminal surface of capillaries in several tissues via heparan sulfate proteoglycans [3–5], and as a head-to-tail homodimer to glycerophosphatidylinositol high-density lipoprotein binding protein-1 [6]. LPL is expressed by several tissues, including adipose, skeletal, cardiac, breast, adrenal tissues, and also macrophages [3,4]. In addition to its catalytic function, LPL also actively facilitates the uptake of lipoproteins and lipid hydrolysis products independently of catalytic activity, via a bridging function that brings the lipoproteins closer to the cell [7].

The expression of LPL is elevated in macrophages residing within atherosclerotic plaques, and its expression is positively correlated to the cholesterol content of the cells [8,9]. In apolipoprotein E-null (*apoE*^*-/-*^) mice, the transgenic expression of human LPL accelerated atherosclerotic lesion formation [10]. Conversely, when *apoE*^*-/-*^ mice were treated with miR-590 (which can inhibit macrophage LPL expression), lesion formation was prevented [11]. In THP-1 macrophages, the lentiviral overexpression of the long non-coding RNA DAPK1-IT1 suppressed miR-590 expression, which in turn increased LPL expression and increased cholesterol accumulation [12]. Though all of the mechanisms by which macrophage LPL contributes to the pathogenesis of atherosclerosis are not yet known, it has roles in the production of pro-inflammatory cytokines, smooth muscle cell recruitment, and it contributes to the lipid uptake of macrophages as part of their transition to lipid-laden foam cells [13–15].

Previous work in our laboratory aimed to characterize the lipid species produced from the hydrolysis of total human lipoproteins by LPL, and it was demonstrated that the LPL hydrolysis products resulted in the phosphorylation of nine major signalling nodes and receptor tyrosine kinases within THP-1 human macrophages after 30 minutes [16]. The treatment of THP-1 macrophages with the total free fatty acid (FFA) component of the LPL hydrolysis products resulted in the phosphorylation of protein kinase B (also named Akt), but none of the other signalling nodes and receptor tyrosine kinases [16]. It was postulated that one or more of the FFA liberated by LPL generated a molecular species of phosphatidylinositol (3,4,5)-trisphosphate (PIP3) that preferentially activated Akt [16]. Akt is a serine/threonine kinase that is downstream of phosphoinositide 3-kinase activity [17,18]. The activation of Akt involves two principal phosphorylation sites: Ser-473 in the regulatory region and Thr-308 in the active site [19]. Akt itself is a kinase that can phosphorylate a variety of downstream proteins with a range of possible functions [18]. For example, Akt phosphorylates tuberous sclerosis factor 2 (TSC2), which inactivates it and prevents it from inhibiting mammalian target of rapamycin complex 1; as a result, the anti-atherogenic process of cholesterol efflux is impaired [20].

We have also previously shown that the hydrolysis products liberated from total lipoproteins by LPL, and notably the total FFA component, impaired the gene expression of the cholesterol efflux transporters ATP binding cassette transporter A1 (ABCA1), ATP binding cassette transporter G1 (ABCG1), and scavenger receptor BI (SR-BI) within THP-1 macrophages after 18 hours [21]; in addition, cholesterol efflux to apolipoprotein A-I (apoA-I) was impaired [21]. Because the total FFA component of lipoprotein hydrolysis products generated by LPL increases the levels of phosphorylated Akt (pAkt) [16] and it inhibits cholesterol efflux [21], and the inhibition of Akt improves cholesterol efflux [20], we suspected that Akt was a key intermediate in the mechanism by which LPL impairs cholesterol efflux. Thus, we hypothesized that one or more specific fatty acids that exist within the total FFA component of lipoprotein hydrolysis products that are generated by LPL impair cholesterol efflux through the activation of Akt. To test this hypothesis, using THP-1 macrophages, we examined the activation of Akt in response to various FFA mixtures that contain the concentrations of FFA species that we previously reported to be found within LPL hydrolysis products from total lipoproteins [16]. We identified that palmitoleate significantly increased Akt phosphorylation. We thus examined cholesterol efflux in response to incubations with palmitoleate and the Akt inhibitor MK-2206. Lastly, we examined the molecular species of phosphoinositides (PIPx) of THP-1 macrophages treated with palmitoleate, to determine if there were changes to select PIPx species that may contribute to a preferential activation of Akt.

## Results

**Fig 1:**
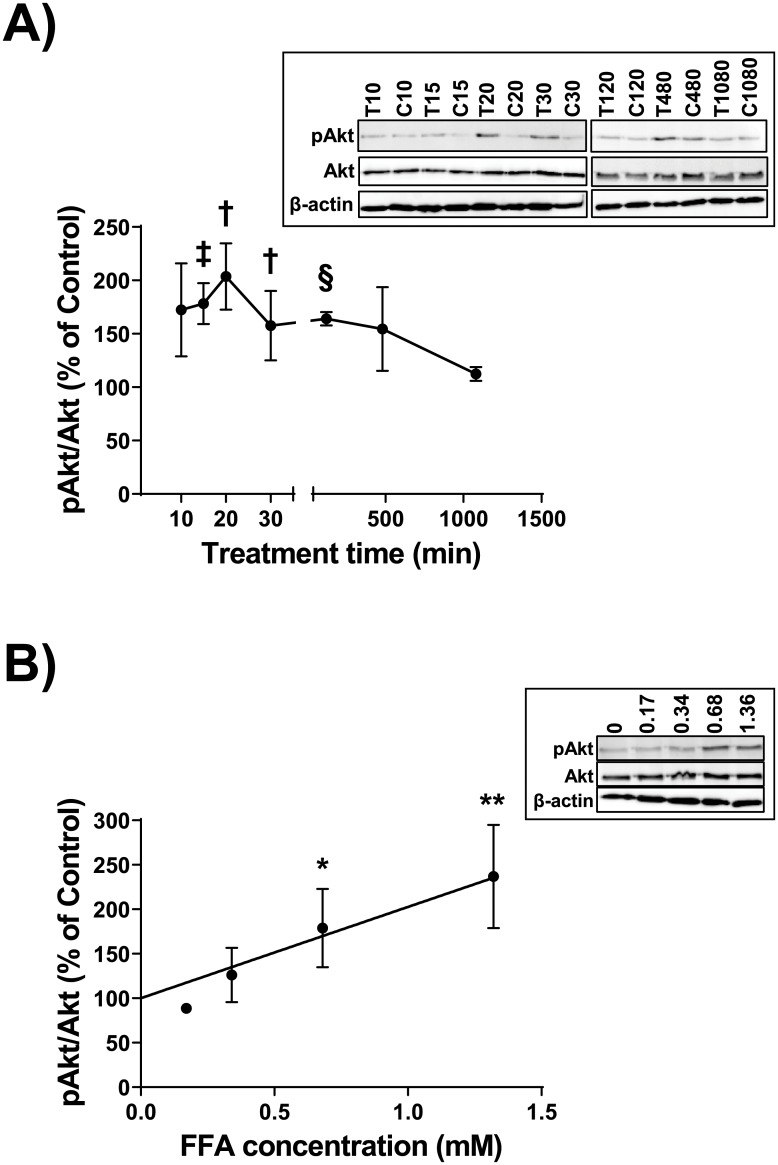
Time course and dose response of Akt phosphorylation. A. THP-1 macrophages were incubated for 0, 10, 15, 20, 30, 120, 420, or 1080 minutes with either a vehicle control (Control or C), or a 0.68 mM mixture of purified FFA (T) that matched the ratios observed post-hydrolysis of total human lipoprotein lipids by LPL. Cell lysates were collected and proteins were subjected to immunoblot analyses. Densitometry of Akt and pAkt were assessed; data are expressed as the ratio of pAkt to total Akt, as a percent of Control. Data are means `SE from three independent experiments, and statistical analysis was performed using multiple t-testing (uncorrected: †, *p* = 0.03; ‡, *p* = 0.008; §, *p* = 0.0003). Following a Bonferroni-Dunn correction (with α = 0.05), only the 120 minute data retained significance (*p* = 0.004). *Inset*, one complete set of immunoblot results from the three independent experiments. The complete set of uncropped immunoblots can be found in S8 Fig. B. THP-1 macrophages were incubated for 2 hours with either a vehicle control (Control), or a mixture of purified FFA that matched the ratios observed post-hydrolysis of total human lipoprotein lipids by LPL; concentrations of 0 mM, 0.17 mM, 0.34 mM, 0.68 mM, and 1.38 mM were tested. Analyses of cell lysates were performed as in “A”. Data are from three independent experiments, and statistical analysis was performed using regression analysis (*R*^*2*^ = 0.49, *p* = 0.01) and a t-test (*, *p* = 0.02; **, *p* = 0.005). *Inset*, one complete set of immunoblot results from the three independent experiments. The complete set of uncropped immunoblots can be found in S9 Fig.

We previously showed using antibody arrays that the hydrolysis products liberated by LPL from total lipoproteins (ρ<1.21 g/ml), as well as the reconstituted total FFA component matching that liberated by LPL at a physiological concentration of 0.68 mM, significantly increased the phosphorylation of Akt after 30 minutes within THP-1 macrophages [16]. In a follow up to these previous observations, we first examined the phosphorylation of Akt within THP-1 macrophages in response to the reconstituted total FFA component matching that liberated by LPL at 0.68 mM, over a time course between 10 minutes and 18 hours. Relative to the vehicle controls for each time point, the percentage of phosphorylation of Ser-473 on Akt was higher, with maximal phosphorylation of 204% observed at 20 minutes (uncorrected *p* = 0.03) (Fig 1A). Following a Bonferroni-Dunn correction on all points that were significant by multiple t-testing alone, only the 2 hour time point retained significance (with corrected *p* = 0.004). Relative to the vehicle controls for each time point, the percentage of phosphorylation of Thr-308 on Akt reached a maximum of 226% at 15 minutes (uncorrected *p* = 0.02) (S1 Fig); however, the phosphorylation returned to control levels by 30 minutes. Following a Bonferroni-Dunn correction on all points that were significant by multiple t-testing alone, none of the time points retained significance. Using the 2 hour time point because it retains significance following correction, we examined the phosphorylation of Akt in response to different concentrations of the FFA mixture. Relative to the vehicle control-treated cells, the percentage of Akt phosphorylation at Ser-473 increased in a dose-dependent fashion with FFA mixture concentrations of 0.17 mM, 0.34 mM, 0.68 mM, and 1.36 mM (Fig 1B).

**Fig 2:**
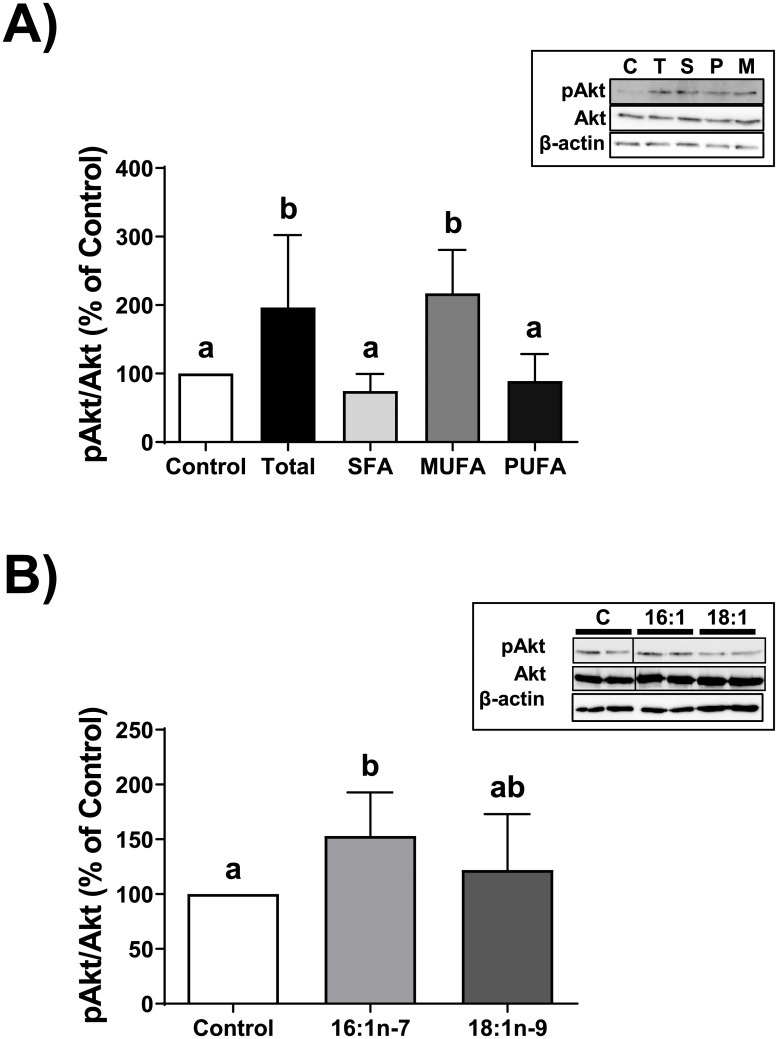
Analysis of FFA classes on Akt phosphorylation. A. THP-1 macrophages were incubated for 2 hours with either a vehicle control (Control or C), a 0.68 mM mixture of purified FFA (Total or T) that matched the ratios observed post-hydrolysis of total human lipoprotein lipids by LPL, the SFA component of the total mixture (S), the MUFA component of the total mixture (M), or the PUFA component of the total mixture (P). Cell lysates were collected and proteins were subjected to immunoblot analyses. Densitometry of Akt and pAkt were assessed; data are expressed as the ratio of pAkt to total Akt, as a percent of Control. Data are from at least five independent experiments. Statistical analysis was performed using a one-way ANOVA with Tukey’s multiple comparisons testing. *Inset*, one complete set of immunoblot results from the five independent experiments. The complete set of uncropped immunoblots can be found in S10 Fig. B. THP-1 macrophages were incubated for 2 hours with either a vehicle control (Control or C), 0.02 mM palmitoleate (16:1n-7 or 16:1), or 0.24 mM oleate (18:1n-9 or 18:1). Cell lysates were collected and proteins were subjected to immunoblot analyses. Analyses of cell lysates and data were performed as in “A”. Data are from five independent experiments. *Inset*, one complete set of immunoblot results from the five independent experiments. The complete set of uncropped immunoblots can be found in S11 Fig.

Having clearly established that Akt phosphorylation was affected by the reconstituted total FFA mixture that matched our previously reported concentrations of FFA species that are liberated by LPL from total lipoproteins, we sought to examine which class of fatty acid within the total FFA mixture might be responsible for the phosphorylation of Akt. We prepared mixtures solely containing the saturated fatty acid (SFA), monounsaturated fatty acid (MUFA), and polyunsaturated fatty acid (PUFA) components of the total FFA mixture, we and tested their ability to phosphorylate Akt within THP-1 macrophages. We chose a 2 hour incubation period because it retains significance following correction (Fig 1A). Our data show that only the MUFA mixture significantly increased the phosphorylation of Akt at Ser-473 by 217% of control levels during a 2 hour period (*p*<0.05), which was comparable to the total FFA mixture (at 196% of control levels) (Fig 2A). We next tested the effect of oleate and palmitoleate, the two components of the MUFA mixture, on the phosphorylation of Akt; we show that only palmitoleate was able to significantly promote the phosphorylation of Akt at Ser-473, by 153% of control levels (*p*<0.05) (Fig 2B).

**Fig 3:**
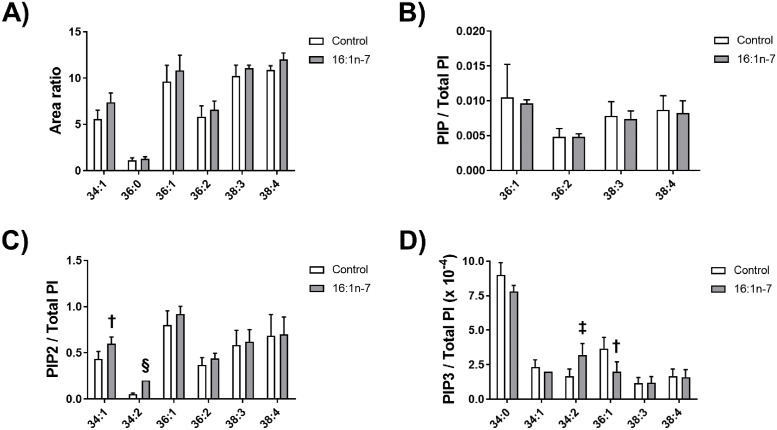
Analyses of PIPx species in response to palmitoleate treatment. THP-1 macrophages were incubated for 18 hours with either a vehicle control (Control) or 0.02 mM palmitoleate (16:1n-7). Lipids were extracted from cell lysates; A, PI species; B, PIP species; C, PIP2 species; and D, PIP3 species were quantified. Data in “A” are expressed as a ratio of area under the curve of the examined species to the area under the curve of the standard PI; data in “B”, “C”, and “D” are expressed as a ratio of intensity per total PI. Control data are from six independent experiments and palmitoleate-treatment data are from five independent experiments. Statistical analysis was performed using a t-test (†, *p* = 0.006; ‡, *p* = 0.005; §, *p*<0.0001).

We previously postulated that the FFA component from lipoprotein hydrolysis products may be generating one or more specific species of PIP3 that preferentially lead to the downstream activation of Akt [16]. Because the MUFA component of the total FFA mixture, and specifically palmitoleate, resulted in the significant phosphorylation of Akt, we examined if palmitoleate would indeed alter the levels of select species of PIPx within THP-1 macrophages over an 18 hour period. We chose this time period because we previously showed it was a sufficient period for the total FFA to impair cholesterol efflux [21]. No significant changes were observed for all assessed species of phosphatidylinositol (PI)–(34:1, 36:0, 36:1, 36:2, 38:3, and 38:4) (Fig 3A), and for all assessed species of phosphatidylinositol monophosphate (PIP)–(36:1, 36:2, 38:3, and 38:4) (Fig 3B). However, there were notable changes in select phosphatidylinositol bisphosphate (PIP2) and PIP3 species. Specifically, compared to control treatment, the treatment with palmitoleate increased the levels of the PIP2 species 34:1 by 138% (*p* = 0.006) and 34:2 PIP2 by 375% (*p*<0.0001) (Fig 3C), while the PIP3 species 36:1 decreased levels to 55% of control (*p* = 0.006) but the PIP3 species 34:2 levels increased by 192% (*p* = 0.005) (Fig 3D).

**Fig 4:**
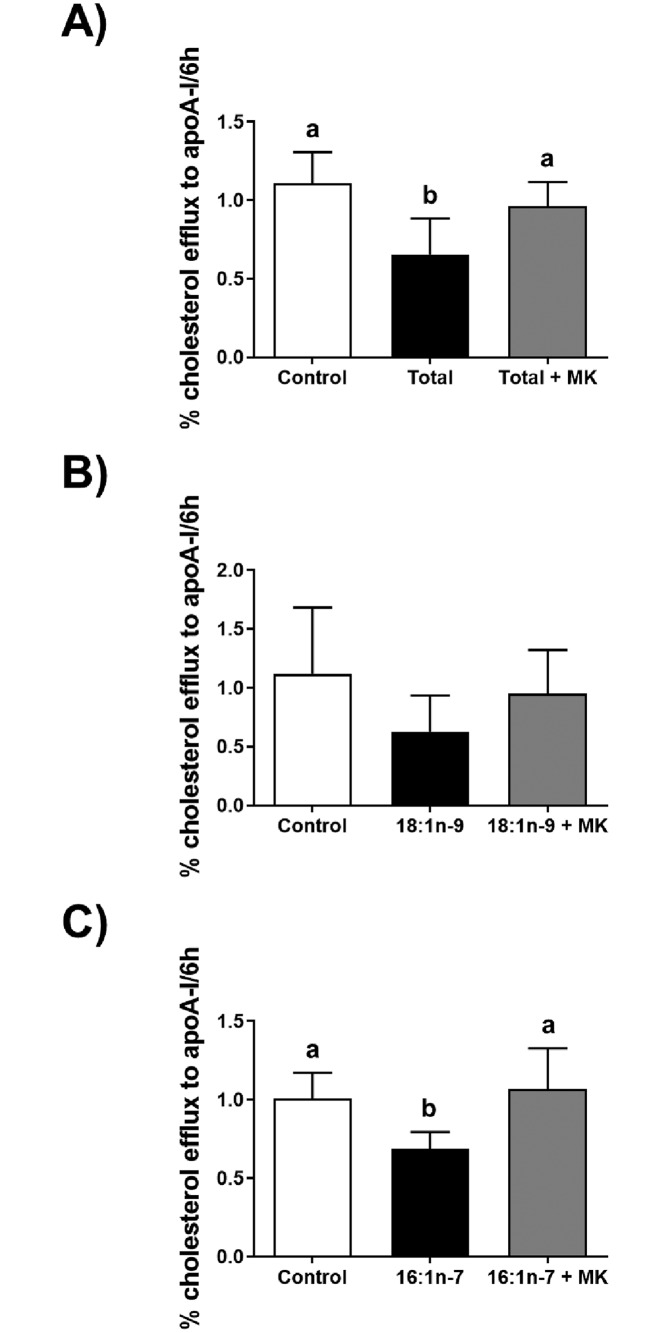
ApoA-I-mediated cholesterol efflux in response to fatty acids and Akt inhibition. THP-1 macrophages labelled with [^3^H]cholesterol were incubated for 18 hours with either a vehicle control (Control); or A, a 0.68 mM mixture of purified FFA (Total) that matched the ratios observed post-hydrolysis of total human lipoprotein lipids by LPL; B, 0.24 mM oleate (18:1n-9); or C, 0.02 mM palmitoleate (16:1n-7). Incubations were in the absence or presence of 1 μM MK-2206 (+ MK). Cholesterol efflux in the absence or presence of 25 μg/ml apoA-I was examined after 6 hours. Cholesterol efflux was calculated as a percent of media [^3^H]cholesterol per total cell and media [^3^H]cholesterol; background efflux (in the absence of apoA-I) was subtracted from efflux data for apoA-I to obtain apoA-I specific efflux. Data in “A” are from eight independent experiments, and data in “B” and “C” are from five independent experiments. Statistical analysis was performed using a one-way ANOVA with Tukey’s multiple comparisons testing.

As we previously showed that the total FFA mixture impaired cholesterol efflux to apoA-I in THP-1 macrophages [21], we sought to determine if impaired cholesterol efflux was in part due to the phosphorylation of Akt. We examined cholesterol efflux to apoA-I in the absence or presence of the Akt-specific inhibitor MK-2206, which abolishes Akt phosphorylation within THP-1 macrophages at a concentration of 1 μM (S2 Fig). Cholesterol efflux to apoA-I was assessed after an 18 hour incubation of cells with the total FFA mixture in the absence or presence of MK-2206, or a vehicle control. As expected, the total FFA treatment reduced the efflux of [^3^H]cholesterol by 35% compared to control levels (*p*<0.001) (Fig 4A). Cholesterol efflux was restored to control levels in the presence of MK-2206. Lastly, we tested cholesterol efflux after an 18 hour incubation with each MUFA component, oleate and palmitoleate, in the absence or presence of MK-2206, or a vehicle control. Of note, oleate was at a 10.2-fold higher concentration versus palmitoleate. While oleate did not significantly affect cholesterol efflux (Fig 4B), palmitoleate significantly reduced cholesterol efflux by 31% compared to control levels (*p*<0.05); again, cholesterol efflux was restored to control levels in the presence of MK-2206. We also examined cholesterol efflux from THP-1 macrophages to HDL, and from acetylated LDL-treated THP-1 macrophages to both apoA-I and HDL, after an 18 hour incubation with palmitoleate; again, palmitoleate significantly reduced cholesterol efflux (S3 Fig). To our surprise, although we previously showed that the total FFA mixture reduced the gene expression of the cholesterol transporters ABCA1, ABCG1, and SR-BI [21], immunoblot analyses showed that the treatment of cells with palmitoleate, or palmitoleate plus MK-2206, did not affect protein expression (S4 Fig).

## Discussion

In this study, we assessed the impact on Akt phosphorylation and cholesterol efflux by the total FFA component that is derived from the hydrolysis of total lipoproteins by LPL. We show that the concentration of palmitoleate that is found within the total FFA component increased Akt phosphorylation and reduced apoA-I-mediated cholesterol efflux (S5 Fig)–an effect that was reversed by inhibiting Akt phosphorylation. The effect on cholesterol efflux to apoA-I and HDL was surprisingly independent of the protein expression for ABCA1, ABCG1, and SR-BI. Collectively, these data point toward an unknown mechanism that negatively influences the anti-atherogenic process of cholesterol efflux.

Akt is a ‘master kinase’ with over 100 unique downstream substrates and clearly defined roles in a variety of functions, such as cell growth and proliferation, insulin response, and angiogenesis [17]. As such, there are a myriad of pathways that could be influenced by a change in Akt phosphorylation status, some of which have implications in atherogenic processes. For example, increased Akt activation within human umbilical vein endothelial cells by tumor necrosis factor-α leads to the increased expression and secretion of monocyte chemoattractant protein-1, a protein involved in the recruitment of monocytes to a developing atherosclerotic lesion area [22]. Akt also phosphorylates TSC2, which in turn prevents it from inhibiting mammalian target of rapamycin complex 1 and ultimately impairing cholesterol efflux; similar to our work, cholesterol efflux was improved in the presence of the Akt inhibitor 10-[4’-(*N*,*N*-diethylamino)butyl]-2-chlorophenoxazine hydrochloride [20]. While *in vitro* studies exist to suggest that Akt phosphorylation may be pro-atherogenic in nature, *in vivo* studies have not provided a clear role for Akt in the development and progression of atherosclerosis. Mice lacking both apolipoprotein E (apoE) and Akt exhibit a more severe atherosclerotic phenotype versus mice lacking only apoE [23]. However, the absence of Akt in low-density lipoprotein receptor-deficient mice did not further influence lesion development [24]. Furthermore, mice lacking apoE display larger atherosclerotic plaque sizes and higher levels of phosphorylated Akt versus mice lacking both apoE and the p110γ subunit of phosphoinositide 3-kinase (PI3K), which prevented Akt phosphorylation [25]. It may be possible that Akt and select downstream pathways play both protective and promotive roles in atherosclerosis, that may be dependent on the stage of development. This will require more careful future examination *in vitro* and *in vivo*.

Perhaps the most interesting finding from our study was that palmitoleate was sufficient to induce the activation of Akt in the THP-1 macrophages and the inhibition of apoA-I- and HDL-mediated cholesterol efflux, while oleate at a 10.2-fold higher concentration failed to significantly affect these processes. We speculated that palmitoleate may reduce the expression of the cholesterol transporters ABCA1, ABCG1, and SR-BI; however, immunoblot analyses showed no changes with transporter expression. Future studies are necessary to examine why HDL-mediated efflux was impaired. Given that the mobilization of free cholesterol from lipid droplets via autophagy upstream of the ABCA1 transporter can be rate-limiting for cholesterol efflux to apoA-I [26,27], one likely possibility is that Akt activation impairs this process. Interestingly, Akt inhibition was shown to increase ABCA1-mediated cholesterol efflux to apoA-I through supressing mammalian target of rapamycin complex 1 (mTORC1) [20]. Conversely, Akt activation by total FFA would be expected to increase mTORC1, which negatively regulates autophagy [28], therefore leading to reduced autophagy-mediated lipid droplet-associated cholesteryl ester catabolism, reduced ABCA1-mediated cholesterol efflux to apoA-I, and cytosolic lipid droplet accumulation. This remains to be investigated. It is possible that other unsaturated fatty acids, such as arachidonate, docosahexaenoate, and eicosapentaenoate, inhibit cholesterol efflux through a similar mechanism, as these have been reported to impede apoA-I-mediated efflux [29]. We failed to show an effect with our PUFA mixture on Akt phosphorylation, but this may be due to differences in concentrations between the latter report and our current study, and potential differences in fatty acid metabolism between cell models.

Although there were no changes with protein expression, we previously reported that *ABCA1*, *ABCG1*, and *SCARB1* mRNA were reduced in response to the total FFA mixture [21]. However, an examination of the expression of the genes encoding these transporters, in the presence of the total FFA mixture without or with the Akt inhibitor MK-2206, showed no difference of expression (S6 Fig). This is consistent with the findings of Huang *et al*. [30], who found that Akt plays no role on the gene expression of cholesterol transporters in the HepG2 human hepatoma cell line. We also previously postulated that specific PIP3 species may be generated to preferentially activate Akt [16]. In the current study, an examination of THP-1 macrophage PIPx in the absence or presence of palmitoleate FFA did reveal differences. Although acyl chain fingerprinting was not performed, the 34:2 species of PIP3 that is increased in THP-1 macrophages treated with palmitoleate may likely represent the incorporation of both palmitoleate and oleate. Also reflecting this increase is the significant increase of the 34:2 PIP2 species. We also observed a significant increase of the 34:1 PIP2 species, which may represent the incorporation of stearate and palmitoleate; this is supported by the modest (but not significant) elevation of the 34:1 PI species. Because the evaluation of PIPx was on samples following an 18 hour incubation, it is possible that an earlier time point may have exhibited a more pronounced increase of select PIPx species in response to palmitoleate, and also a likely elevation of 34:1 PIP3, which we did not observe. However, while there remains more to elucidate about the mechanism of activation, these data provide clues to specific species of PIPx that may preferentially influence the PI3K-Akt pathway and cholesterol efflux–a focus of future investigation that is beyond the scope of the current study.

In a 2009 meta-analysis evaluating the effects of certain types of fats on cardiovascular outcomes, it was suggested that replacing the SFA in the diet with PUFA or MUFA could improve outcomes [31]. The authors’ hypothesis was initially that either MUFA or PUFA could improve cardiovascular disease risk when compared to SFA, but they surprisingly found no association with MUFA. The authors indicated that the source of dietary MUFA was primarily derived from animal fat, and they suggested that it was a possible confounding factor in their conclusions [31]. However, our data suggest that MUFA incorporation into atherosclerotic plaques may actually be an accelerating factor by partially inhibiting cholesterol efflux. In fact, the rate of degradation of ABCA1 was shown to be increased in two different mouse macrophage models in the presence of unsaturated fatty acids [32]. What remains to be tested is how the impaired cholesterol efflux in response to a palmitoleate-enriched diet would translate into reverse cholesterol transport functionality *in vivo*.

In conclusion, we have demonstrated that palmitoleate increases Akt phosphorylation in THP-1 macrophages and reduces apoA-I-mediated cholesterol efflux through a mechanism mediated by Akt. It is possible that other macrophage cell lines that do not require phorbol 12-myristate 13-acetate (PMA) for differentiation exhibit the same phenomenon, but this remains to be examined. The likely accumulation of sterols in response to the total FFA mixture is further supported by work in our laboratory showing that the inhibition of PI3K reduces FFA-mediated neutral lipid accumulation in our model (S7 Fig). Of note, the total FFA mixture we used was derived from the concentration of FFA species that we previously reported are from total lipoproteins in the presence of LPL. These lipoproteins, and our subsequently tested FFA concentrations, were based on lipoproteins isolated from normolipidemic subjects. In the future, it would be of interest to examine the response of the PI3K-Akt pathway using lipoproteins, and the FFA component liberated by LPL, from subjects with controlled and uncontrolled hyperlipidemia; samples from such subjects might exhibit unique lipoprotein lipidomes that may impair cholesterol efflux in response to LPL hydrolysis, in addition to harboring high-density lipoproteins with poor cholesterol efflux capacity.

## Materials and methods

### Preparation of free fatty acid mixtures

A total FFA mixture matching concentrations that we previously reported to be liberated by LPL during hydrolysis of total lipoproteins was prepared as previously described [16,21]. Briefly, myristate, palmitoleate, palmitate, linoleate, oleate, stearate, arachidonate, and docosahexaenoate (all purchased from Nu-Chek Prep, Elysian, MN, USA) were stored until needed at -20°C under N_2(g)_ as 10 mg/ml solutions in liquid chromatography-mass spectrometry grade methanol (Fisher Scientific, Ottawa, ON, Canada). Prior to preparation of FFA mixtures, stock FFA were brought to room temperature. To prepare 1 ml media with 0.68 mM total FFA for cell culture, 18.6 nmol myristate, 23.7 nmol palmitoleate, 275.0 nmol palmitate, 70.0 nmol linoleate, 241.8 nmol oleate, 45.4 nmol stearate, 0.9 nmol arachidonate, and 0.4 nmol docosahexaenoate were removed from stock solutions and the methanol evaporated at 35°C under N_2(g)_; the fatty acids were resuspended in 30 μl dimethylsulfoxide. The fatty acid/dimethylsulfoxide mixture or 30 μl dimethylsulfoxide (as vehicle control) was added at a rate of 1 μl/min to 990 μl RPMI-1640 (cat. #SH30255.01, Fisher Scientific) medium containing 0.2% w/v fatty acid-free bovine serum albumin (FAF-BSA, Millipore Sigma, St. Louis, MO, USA), 1% v/v antibiotic/antimycotic (A/A, Fisher Scientific), and 100 nM PMA (Millipore Sigma) while continuously vortexing. Media containing only SFA (18.6 nmol myristate, 275.0 nmol palmitate, and 45.4 nmol stearate), only MUFA (23.7 nmol palmitoleate and 241.8 nmol oleate), and only PUFA (70.0 nmol linoleate, 0.9 nmol arachidonate, and 0.4 nmol docosahexaenoate), were similarly prepared. The FFA mixtures were used to treat THP-1 macrophages (described below).

### Treatment of THP-1 macrophages with FFA mixtures

THP-1 human monocytic leukemia cells (American Type Culture Collection, Manassas, VA, USA) were cultured in T75 flasks with RPMI, which was further supplemented with 10% v/v fetal bovine serum (cat. #SH30396.03, Fisher Scientific) and 1% v/v A/A. The cells were incubated at 37°C with 5% CO_2(g)_. To differentiate cells into macrophages, THP-1 cells were seeded into each well of a 6-well plate at a density of 3.86 x 10^5^ cells/ml in RPMI containing 10% v/v fetal bovine serum, 1% v/v A/A, and 100 nM PMA. After 48 hours, cells were washed three times with RPMI, then incubated with RPMI containing 0.2% w/v FAF-BSA, 1% v/v A/A, and 100 nM PMA. After 24 hours, cells were washed three times with RPMI, then incubated for 2 hours (unless otherwise stated) with 1 ml of media containing FFA (prepared as described above), or the vehicle control media. Following incubation, cells were washed three times with 2 ml ice-cold phosphate-buffered saline (pH 7.0). Cells were lysed on ice for 15 minutes with a commercial buffer (#98038, Cell Signaling Technology, Danvers, MA, USA), supplemented with 0.1% v/v protease/phosphatase inhibitor (#5872S, Cell Signaling Technology); cells were collected and stored at -80°C until needed. Prior to use, the protein content of cell lysates was quantified using a bicinchonic acid protein assay kit (Fisher Scientific), according to manufacturer’s instructions.

### Immunoblotting

Proteins (5 μg) were separated by SDS-PAGE using 12% resolving gels and transferred to nitrocellulose membranes. For ABCA1, 60 μg of protein were separated by SDS-PAGE using 8% resolving gels. Following transfer, the membranes were blocked overnight at 4°C in Tris-buffered saline (TBS) at pH 7.4, containing 5% w/v bovine serum albumin (BSA, Millipore Sigma), 0.05% v/v Tween-20 (Fisher Scientific), and 0.05% w/v NaN_3_ (Fisher Scientific). After blocking, the membranes were incubated at 4°C overnight with antibodies against human Akt (1:1,000 dilution of cat. #9272, Cell Signaling Technology), Ser-473 phosphorylated human Akt (1:2,000 dilution of cat. #4060, Cell Signaling Technology), Thr-308 phosphorylated human Akt (1:2,000 dilution of cat. #2965S, Cell Signaling Technology), human ABCA1 (1:500 dilution of cat. #NB400-105, Novus Biologicals, Centennial, CO, USA), human ABCG1 (1:1,000 dilution of cat. #NB400-132, Novus Biologicals), human SR-BI (1:500 dilution of cat. #NB400-104, Novus Biologicals), or human β-actin (1:5,000 dilution of cat. #NB600-501, Novus Biologicals). All primary antibodies were diluted with TBS containing 5% BSA, 0.05% v/v Tween-20, 0.05% w/v NaN_3_. Following incubation, membranes were washed four times with TBS containing 0.05% v/v Tween-20, then incubated for 2 hours with horseradish peroxidase-conjugated antibodies against rabbit IgG to detect Akt, pAkt, ABCA1, ABCG1, and SR-BI (1:2,000 dilution of cat. #SA1-200, Fisher Scientific) or mouse IgG to detect β-actin (1:2,000 dilution of cat. #SA1-100, Fisher Scientific). Following incubation, membranes were washed four times with TBS, then visualized using an ImageQuant LAS 4000 system (GE Healthcare, Baie d’Ufre, QC, Canada) following development using the ECL^™^ Prime Western Blotting Detection Reagent (GE Healthcare), according to manufacturer’s protocol. The densitometry of protein bands was quantified using ImageJ [33].

### Cholesterol efflux assays

ApoA-I (Lee Biosolutions, Maryland Heights, MO, USA) was salt-exchanged using a PD-10 desalting column (GE Healthcare) equilibrated with phosphate-buffered saline, and the protein concentration was measured using the absorbance at 280 nm and the molar absorption coefficient of 1.23 ml/ mg·cm [34]. Cholesterol efflux assays using THP-1 macrophages were carried out as previously described [21]. In brief, cells were cultured in 12-well plates and radiolabelled with 1 μCi/ml [^3^H]cholesterol (PerkinElmer, Waltham, MA, USA). Following labelling, cells were incubated for 18 hours with media containing either fatty acid (0.68 mM total fatty acid mixture, 23.7 μM palmitoleate, or 241.8 μM oleate) or vehicle control, in the absence or presence of 1 μM the Akt inhibitor MK-2206 (Selleckchem, Houston, TX, USA). After incubation without or with fatty acid, cells were incubated without or with 25 μg/ml apoA-I for 6 hours. After 6 hours, media were collected and cells were lysed using 1 ml of 0.2 M NaOH for 30 minutes; [^3^H]cholesterol from media and cell lysates were quantified by scintillation counting. The amount of [^3^H]cholesterol effluxed was calculated as a percentage of [^3^H]cholesterol effluxed into the medium per amount of total cell and medium [^3^H]cholesterol. Background efflux (in the absence of apoA-I) was subtracted from efflux data to obtain apoA-I specific efflux.

### Mass spectrometry

PI, PIP, PIP2, and PIP3 were analyzed as previously described [35]. In brief, lipids were extracted from cell pellets with acidified solvents prior to derivatization with diazomethyltimethylsilane. Dry derivatized lipid extracts were dissolved in 100 μl methanol (80% aqueous solution) prior to liquid chromatography separation with mass spectrometry detection using an Acquity UPLC system system hyphenated to a QTRAP 4000 mass spectrometer (Waters, Wilmslow, U.K). The mobile phase consisted of a linear gradient from 45% acetonitrile with 0.1% formic acid in water, changing over 20 minutes to 90% acetonitrile with 0.1% of formic acid in water. The column used was a Waters BEH 300 C4 (100 × 1.0 mm), 1.7 μm. The areas of the total ion current of each PIP, PIP2 and PIP3 lipid molecular species were corrected for recovery to internal standard. The reported data were normalized to the recovery of the PI content.

### Statistical analyses

Statistical analyses were performed using GraphPad Prism 8.2. An unpaired Student’s t test or one-way ANOVA with Tukey’s multiple comparison post-test were used, unless otherwise stated. A *p*<0.05 was considered significantly different. Unless otherwise stated, all data are given as mean ± SD.

## Supporting information

S1 Fig Time course of Thr-308 phosphorylation of Akt. THP-1 macrophages were incubated for 0, 10, 15, 20, 30, 120, 420, or 1080 minutes with either a vehicle control (Control or C), or a 0.68 mM mixture of purified FFA (T) that matched the ratios observed post-hydrolysis of total human lipoprotein lipids by LPL. Densitometry of Akt and pAkt were assessed; data are expressed as the ratio of pAkt to total Akt, as a percent of Control. Data are means ± SE from three independent experiments, and statistical analysis was performed using multiple t-testing (*, *p* = 0.02; **, *p* = 0.01). Following a Bonferroni-Dunn correction (with α = 0.05), no points retained significance. *Inset*, one complete set of immunoblot results from the three independent experiments. The complete set of uncropped immunoblots can be found in S8 Fig.
(PDF)

S2 Fig Phosphorylation of Akt in the absence or presence of MK-2206. THP-1 macrophages were incubated with a DMSO vehicle control (C) or with 0.5, 1, or 2 μM MK-2206 for 18 hours. A. Immunoblot of pAkt (Ser-473). B. Immunoblot of Akt. Images are representative of technical replicates from one experiment.
(PDF)

S3 Fig ApoA-I- and HDL-mediated cholesterol efflux in response to palmitoleate treatment of THP-1 macrophages without or with acetylated LDL loading. THP-1 macrophages labelled with [^3^H]cholesterol in the absence (Unloaded) or presence of 50 μg/ml acetylated LPL (acLDL) were incubated for 18 hours with either a vehicle control (Control) or 0.02 mM palmitoleate (PA). Cholesterol efflux in the absence or presence of 50 μg/ml apoA-I or 50 μg/ml HDL was examined after 6 hours. Cholesterol efflux was calculated as a percent of media [^3^H]cholesterol per total cell and media [^3^H]cholesterol; background efflux (in the absence of apoA-I or HDL) was subtracted from efflux data for apoA-I (or HDL) to obtain apoA-I (or HDL) specific efflux. Data are means ± standard error, from a representative experiment with triplicate wells. Statistical analysis was performed using a t-test.
(PDF)

S4 Fig Protein expression of ABCA1, ABCG1, and SR-BI. In three independent experiments, THP-1 macrophages were incubated for 18 hours with either a vehicle control (Control), 0.02 mM palmitoleate (16:1 or 16:1n-7), or 0.02 mM palmitoleate in the presence of 1 μM MK-2206 (+ MK). Cell lysates were collected and proteins were subjected to immunoblot analyses. A. One complete set of immunoblot results. The complete set of uncropped immunoblots can be found in S12 Fig. B. Densitometry analysis of ABCA1, expressed as a percent of Control. C. Densitometry analysis of ABCG1, expressed as a percent of Control. D. Densitometry analysis of ABCA1, expressed as a percent of Control. Note, data were normalized to densitometry data for β-actin.
(PDF)

S5 Fig Model of palmitoleate influence on Akt and cholesterol efflux. In our model, macrophages take up the palmitoleate introduced to the cell medium through a fatty acid transporter (likely CD36). We suspect that palmitoleate can then be incorporated into phosphatidylinositide biosynthesis and leading to a PIP3 species (via phosphatidylinositol 3-kinase (PI3K)) that preferentially activates both phosphoinositide-dependent kinase 1 (PDK1) and the mammalian target of rapamycin (mTOR) complex 2 (mTORC2—which includes mTOR, mammalian lethal with sec13 protein (mLST8), Rictor, and stress-activated protein kinase-interacting protein 1 (SIN1)). At the membrane interface, Akt is then in turn phosphorylated by PDK1 at Thr308 and by mTORC2 at Ser473. The phosphorylation of Akt renders it active, allowing it to phosphorylate other proteins. The Akt-mediated pathway influencing cholesterol efflux remains to be determined.
(PDF)

S6 Fig Expression of *ABCA1*, *ABCG1*, and *SCARB1* mRNA in the presence of the total FFA mixture without or with MK-2206. THP-1 macrophages were treated with either 0.68 mM of the total FFA mixture (Total), or 0.68 mM FFA with 1 μM MK-2206 (Total + MK) for 18 hours. RNA was collected, and real-time PCR was performed on the samples using primers for A, *ABCA1*; B, *ABCG1*; and C, *SCARB1*. Data are from four independent experiments, and are presented as a percentage of FFA alone, normalized to β-actin.
(PDF)

S7 Fig Oil Red O staining of total FFA-treated THP-1 macrophages in the absence or presence of LY294002 (LY). THP-1 macrophages were incubated with the total FFA mixture (0.68 mM) for 18 hours. Cells were subsequently stained with Oil red O, and the stain was extracted using isopropanol and quantified at 520 nm. Data are from six independent experiments.
(PDF)

S8 Fig Uncropped immunoblot images comprising those from Fig 1A and S1 Fig. THP-1 macrophages were incubated for 0, 10, 15, 20, 30, 120, 420, or 1080 minutes with either a vehicle control (Control or C), or a 0.68 mM mixture of purified FFA (T) that matched the ratios observed post-hydrolysis of total human lipoprotein lipids by LPL. Cell lysates were collected and proteins were subjected to immunoblot analyses. Shown are one complete set of immunoblot results. A. pAkt (of Ser-473) at 10, 15, 20, and 30 minutes. B. pAkt (of Ser-473) at 120, 480, and 1080 minutes. C. pAkt (of Thr-308) at 10, 15, 20, and 30 minutes. D. pAkt (of Thr-308) at 120, 480, and 1080 minutes. E. Akt at 10, 15, 20, and 30 minutes. F. Akt at 120, 480, and 1080 minutes. G. β-actin at 10, 15, 20, and 30 minutes. H. β-actin at 120, 480, and 1080 minutes.
(PDF)

S9 Fig Uncropped immunoblot images comprising those from Fig 1B. THP-1 macrophages were incubated for 2 hours with either a vehicle control (Control), or a mixture of purified FFA that matched the ratios observed post-hydrolysis of total human lipoprotein lipids by LPL; concentrations of 0 mM, 0.17 mM, 0.34 mM, 0.68 mM, and 1.38 mM were tested. Cell lysates were collected and proteins were subjected to immunoblot analyses. Shown are one complete set of immunoblot results. A. pAkt (of Ser-473). B. Akt. C. β-actin.
(PDF)

S10 Fig Uncropped immunoblot images comprising those from Fig 2A. THP-1 macrophages were incubated for 2 hours with either a vehicle control (Control or C), a 0.68 mM mixture of purified FFA (Total or T) that matched the ratios observed post-hydrolysis of total human lipoprotein lipids by LPL, the SFA component of the total mixture (S), the MUFA component of the total mixture (M), or the PUFA component of the total mixture (P). Cell lysates were collected and proteins were subjected to immunoblot analyses. A. pAkt (of Ser-473). B. Akt. C. β-actin.
(PDF)

S11 Fig Uncropped immunoblot images comprising those from Fig 2B. THP-1 macrophages were incubated for 2 hours with either a vehicle control (Control or C), 0.02 mM palmitoleate (16:1n-7 or 16:1), or 0.24 mM oleate (18:1n-9 or 18:1). Cell lysates were collected and proteins were subjected to immunoblot analyses. A. pAkt (of Ser-473). B. Akt. C. β-actin. Note, “A” and “B” also include the total FFA mixture at 0.68 mM but these data were not part of Fig 2B.
(PDF)

S12 Fig Uncropped immunoblot images comprising those from S4 Fig. THP-1 macrophages were incubated for 18 hours with either a vehicle control (Control), 0.02 mM palmitoleate (16:1), or 0.02 mM palmitoleate in the presence of 1 μM MK-2206 (+ MK). Cell lysates were collected and proteins were subjected to immunoblot analyses. A. ABCA1. B. ABCG1. C. SR-BI. D. β-actin. Note, lanes labelled with red text were used for S4 Fig.
(PDF)

S1 File Supporting materials and methods. Materials and methods associated with S3, S6 and S7 Figs.
(PDF)
