# Supplementary figures and images for: THP-1 macrophage cholesterol efflux is impaired by palmitoleate through Akt activation
Source: PLoS One. 2020 May 21;15(5):e0233180. doi: 10.1371/journal.pone.0233180 (PMC7241781; doi:10.1371/journal.pone.0233180)

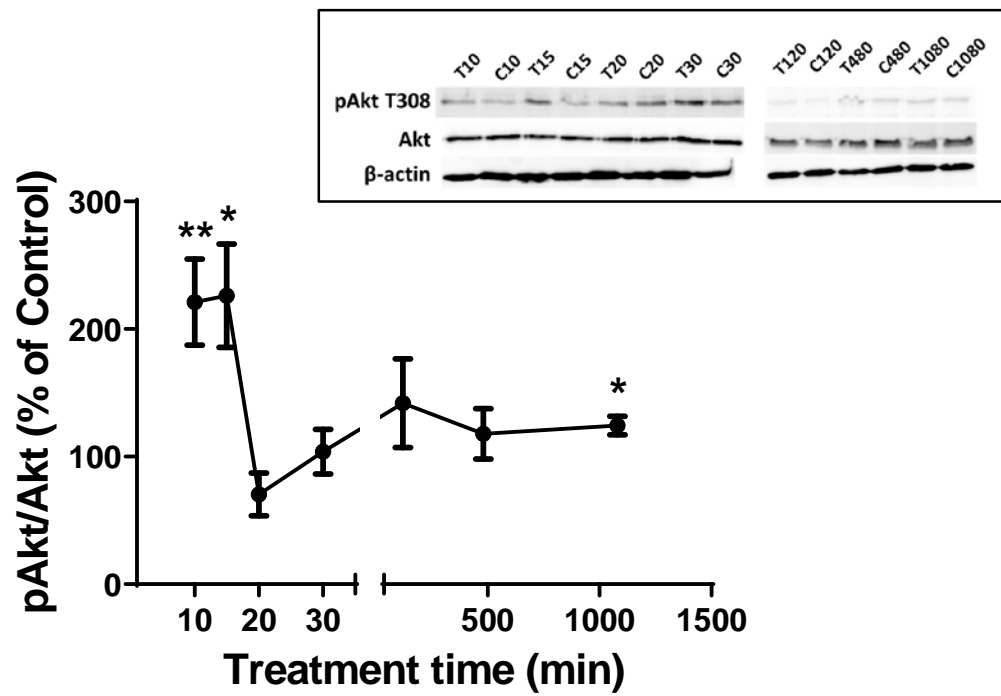

Supplement: S1 Fig — THP-1 macrophages were incubated for 0, 10, 15, 20, 30, 120, 420, or 1080 minutes with either a vehicle control (Control or C), or a 0.68 mM mixture of purified FFA (T) that matched the ratios observed post-hydrolysis of total human lipoprotein lipids by LPL. Densitometry of Akt and pAkt were assessed; data are expressed as the ratio of pAkt to total Akt, as a percent of Control. Data are means ± SE from three independent experiments, and statistical analysis was performed using multiple t-testing (*, p = 0.02; **, p = 0.01). Following a Bonferroni-Dunn correction (with α = 0.05), no points retained significance. Inset, one complete set of immunoblot results from the three independent experiments. The complete set of uncropped immunoblots can be found in S8 Fig. (PDF) [file pone.0233180.s001.pdf]

**A)**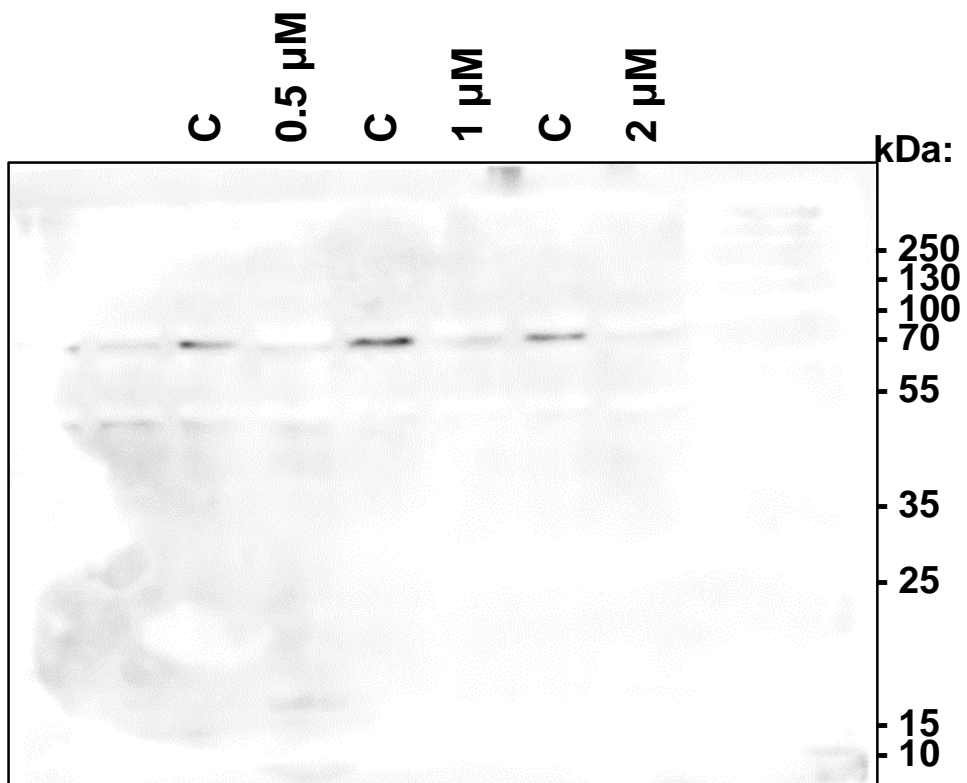**B)**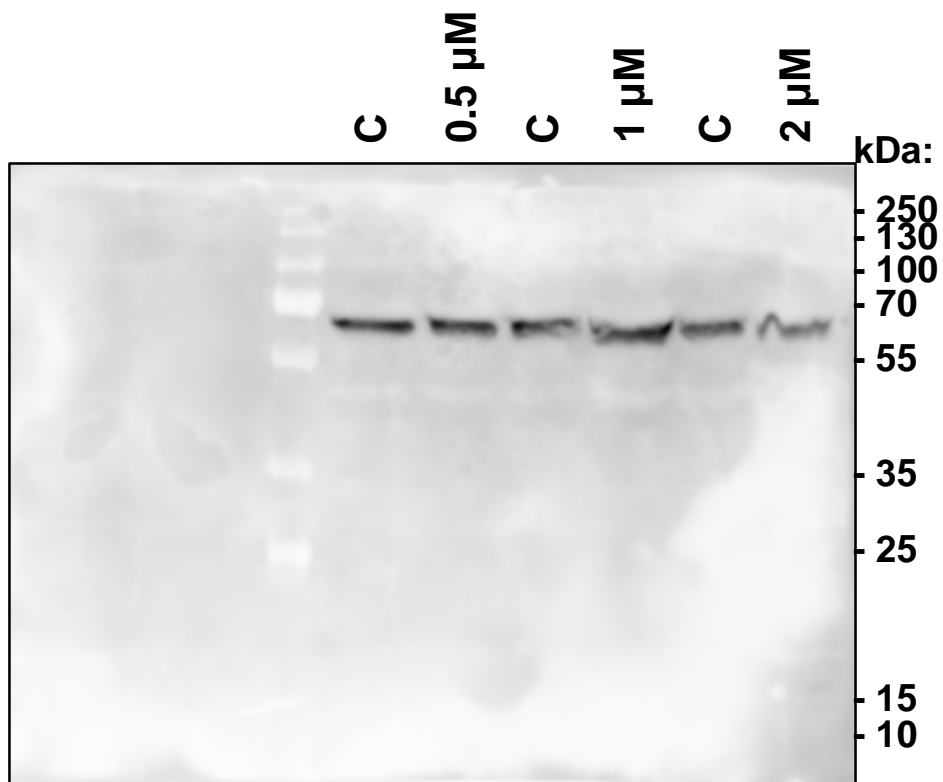

Supplement: S2 Fig — THP-1 macrophages were incubated with a DMSO vehicle control (C) or with 0.5, 1, or 2 μM MK-2206 for 18 hours. A. Immunoblot of pAkt (Ser-473). B. Immunoblot of Akt. Images are representative of technical replicates from one experiment. (PDF) [file pone.0233180.s002.pdf]

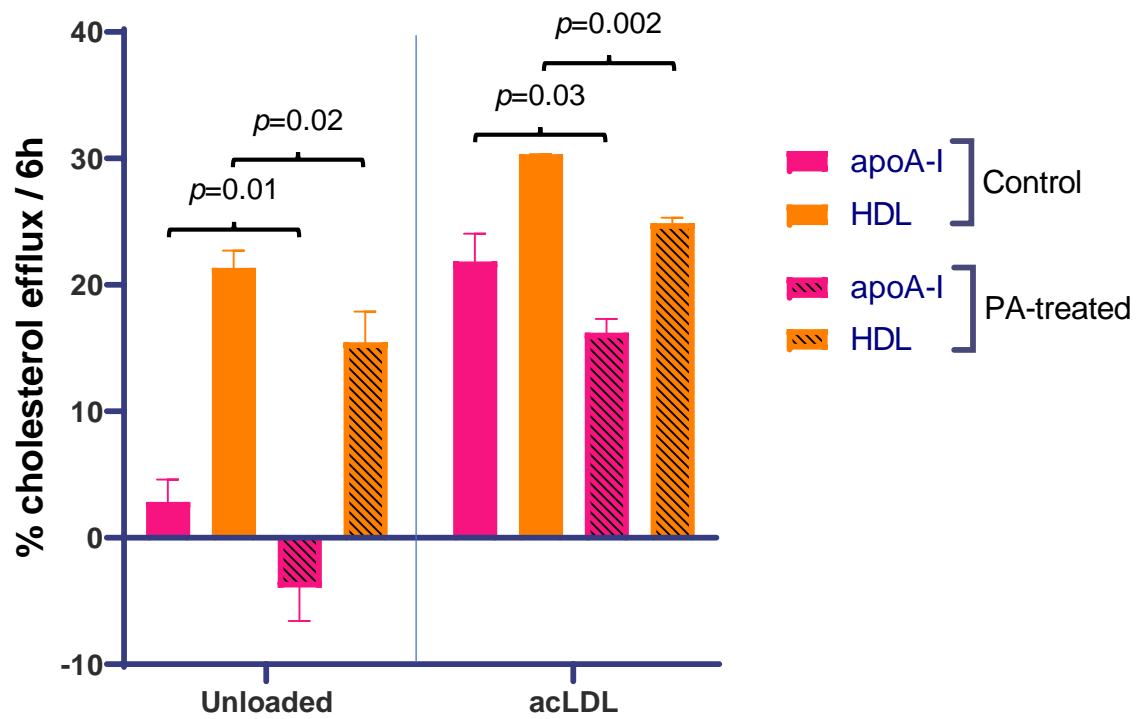

Supplement: S3 Fig — THP-1 macrophages labelled with [3H]cholesterol in the absence (Unloaded) or presence of 50 μg/ml acetylated LPL (acLDL) were incubated for 18 hours with either a vehicle control (Control) or 0.02 mM palmitoleate (PA). Cholesterol efflux in the absence or presence of 50 μg/ml apoA-I or 50 μg/ml HDL was examined after 6 hours. Cholesterol efflux was calculated as a percent of media [3H]cholesterol per total cell and media [3H]cholesterol; background efflux (in the absence of apoA-I or HDL) was subtracted from efflux data for apoA-I (or HDL) to obtain apoA-I (or HDL) specific efflux. Data are means ± standard error, from a representative experiment with triplicate wells. Statistical analysis was performed using a t-test. (PDF) [file pone.0233180.s003.pdf]

**A)**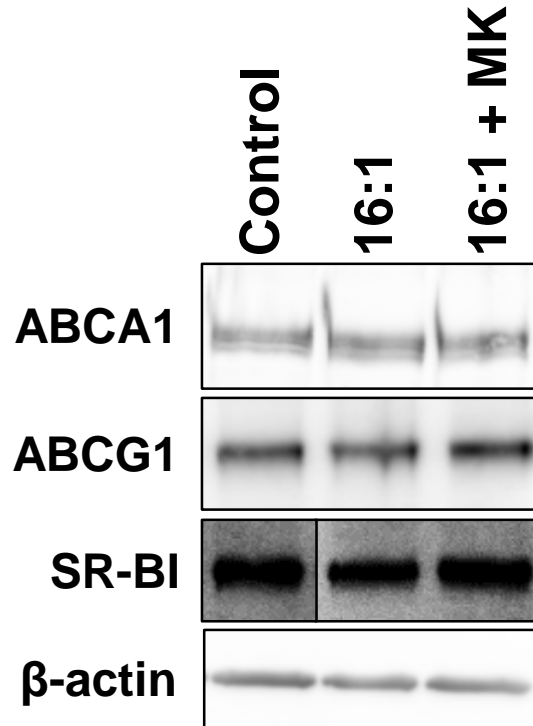**B)**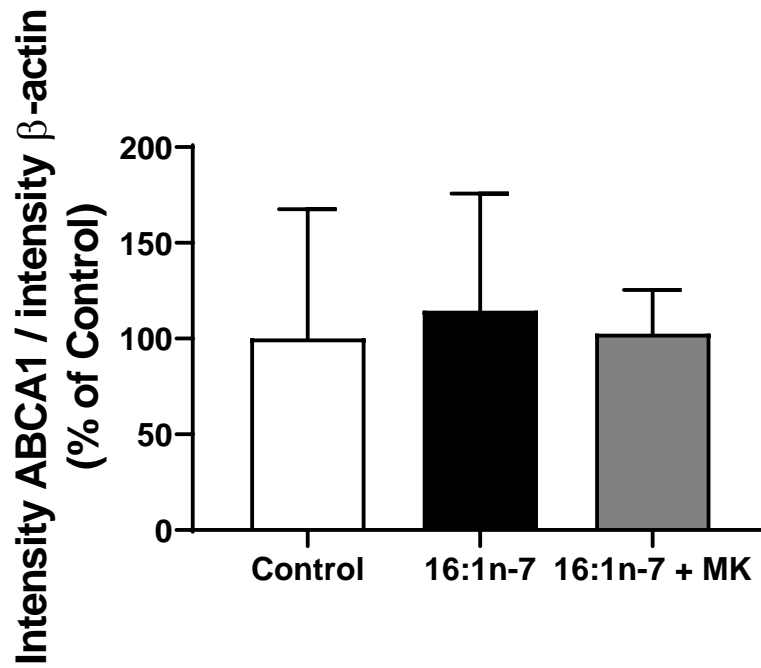

C)

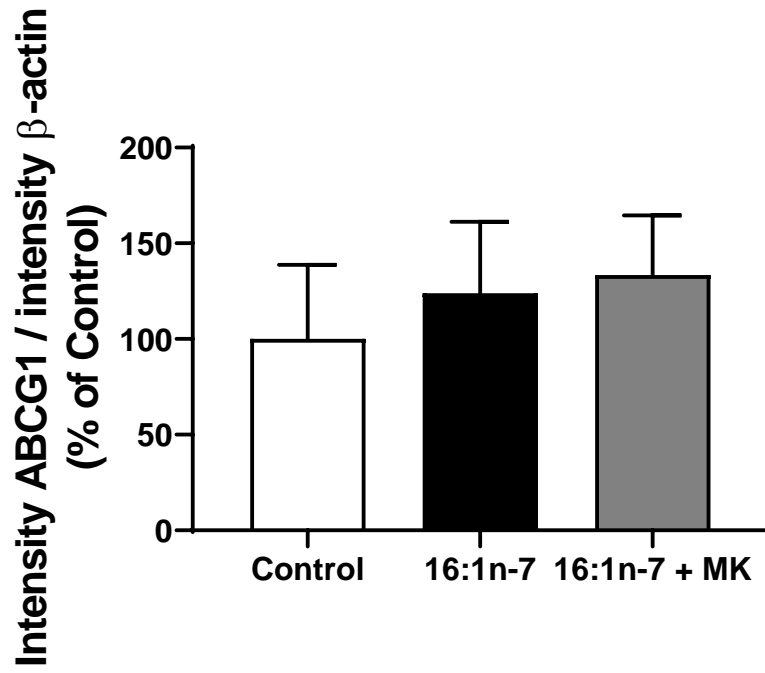

D)

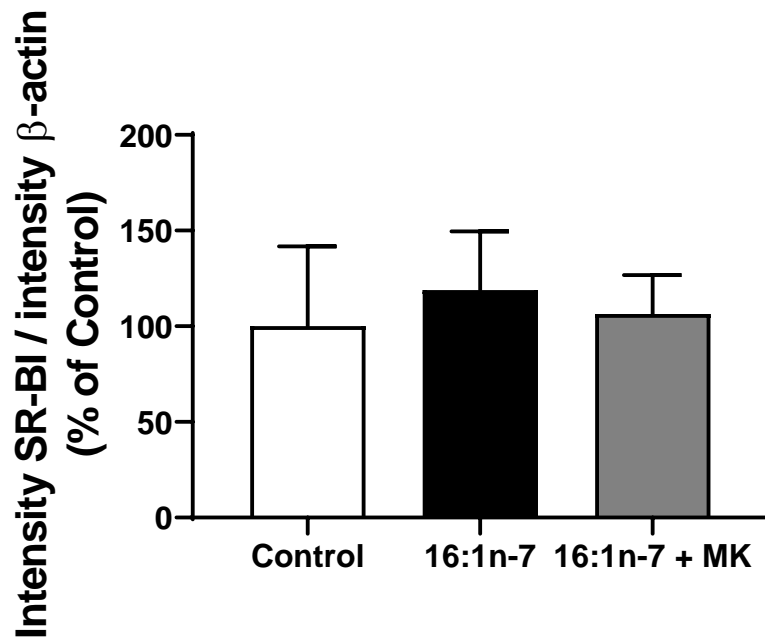

Supplement: S4 Fig — In three independent experiments, THP-1 macrophages were incubated for 18 hours with either a vehicle control (Control), 0.02 mM palmitoleate (16:1 or 16:1n-7), or 0.02 mM palmitoleate in the presence of 1 μM MK-2206 (+ MK). Cell lysates were collected and proteins were subjected to immunoblot analyses. A. One complete set of immunoblot results. The complete set of uncropped immunoblots can be found in S12 Fig. B. Densitometry analysis of ABCA1, expressed as a percent of Control. C. Densitometry analysis of ABCG1, expressed as a percent of Control. D. Densitometry analysis of ABCA1, expressed as a percent of Control. Note, data were normalized to densitometry data for β-actin. (PDF) [file pone.0233180.s004.pdf]

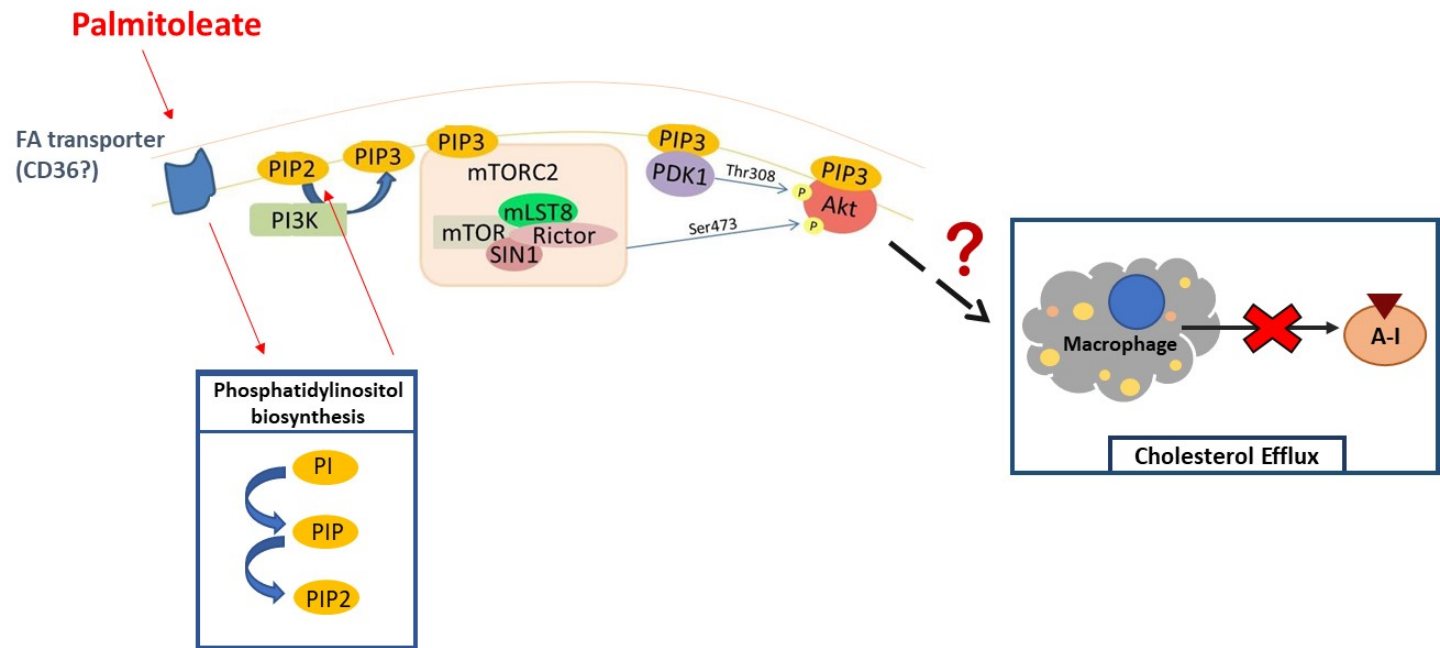

Supplement: S5 Fig — In our model, macrophages take up the palmitoleate introduced to the cell medium through a fatty acid transporter (likely CD36). We suspect that palmitoleate can then be incorporated into phosphatidylinositide biosynthesis and leading to a PIP3 species (via phosphatidylinositol 3-kinase (PI3K)) that preferentially activates both phosphoinositide-dependent kinase 1 (PDK1) and the mammalian target of rapamycin (mTOR) complex 2 (mTORC2—which includes mTOR, mammalian lethal with sec13 protein (mLST8), Rictor, and stress-activated protein kinase-interacting protein 1 (SIN1)). At the membrane interface, Akt is then in turn phosphorylated by PDK1 at Thr308 and by mTORC2 at Ser473. The phosphorylation of Akt renders it active, allowing it to phosphorylate other proteins. The Akt-mediated pathway influencing cholesterol efflux remains to be determined. (PDF) [file pone.0233180.s005.pdf]

**A)**

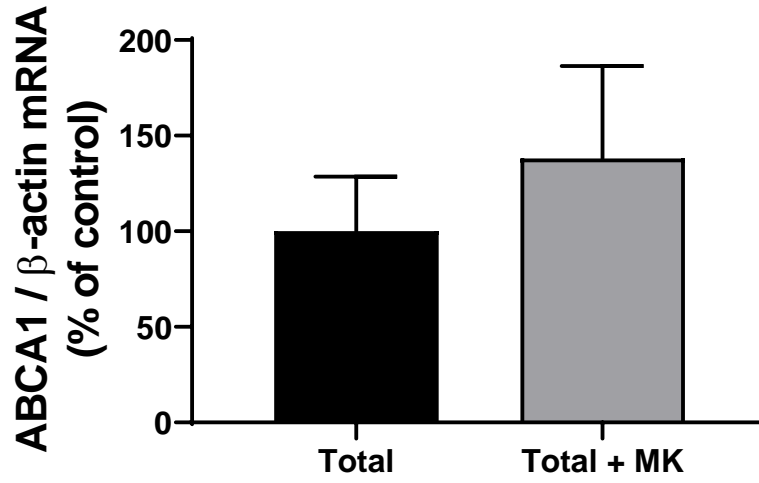

**B)**

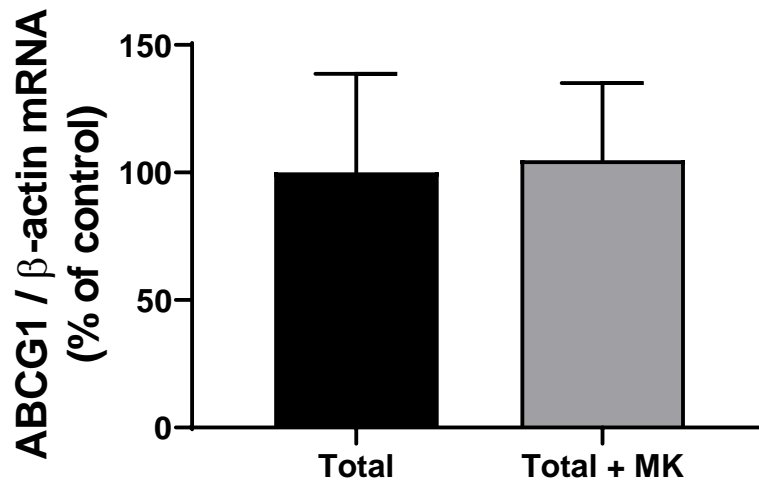

C)

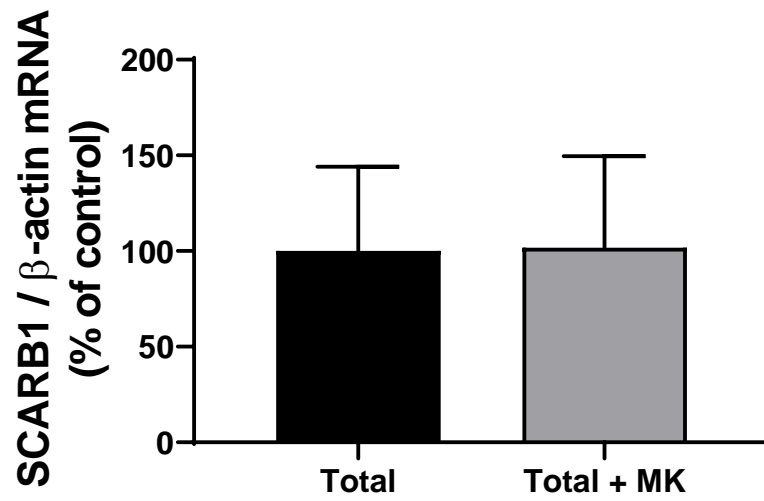

Supplement: S6 Fig — THP-1 macrophages were treated with either 0.68 mM of the total FFA mixture (Total), or 0.68 mM FFA with 1 μM MK-2206 (Total + MK) for 18 hours. RNA was collected, and real-time PCR was performed on the samples using primers for A, ABCA1; B, ABCG1; and C, SCARB1. Data are from four independent experiments, and are presented as a percentage of FFA alone, normalized to β-actin. (PDF) [file pone.0233180.s006.pdf]

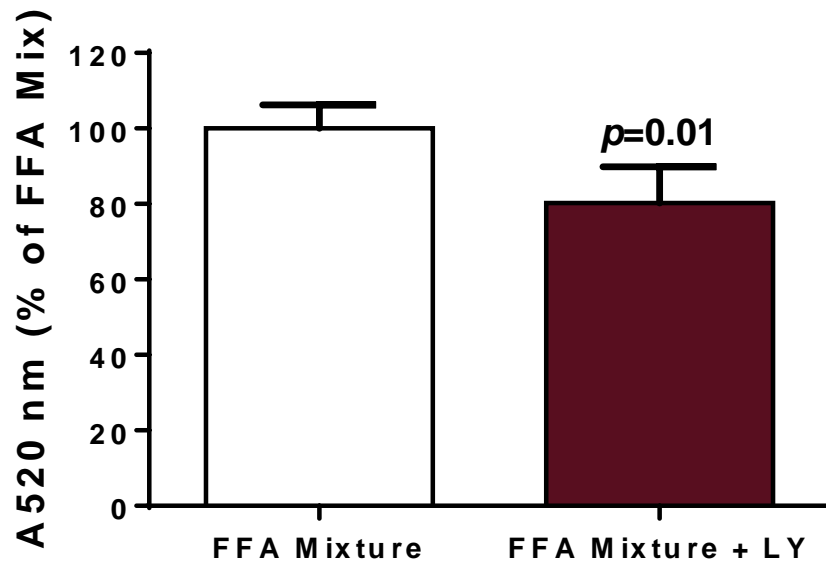

Supplement: S7 Fig — THP-1 macrophages were incubated with the total FFA mixture (0.68 mM) for 18 hours. Cells were subsequently stained with Oil red O, and the stain was extracted using isopropanol and quantified at 520 nm. Data are from six independent experiments. (PDF) [file pone.0233180.s007.pdf]

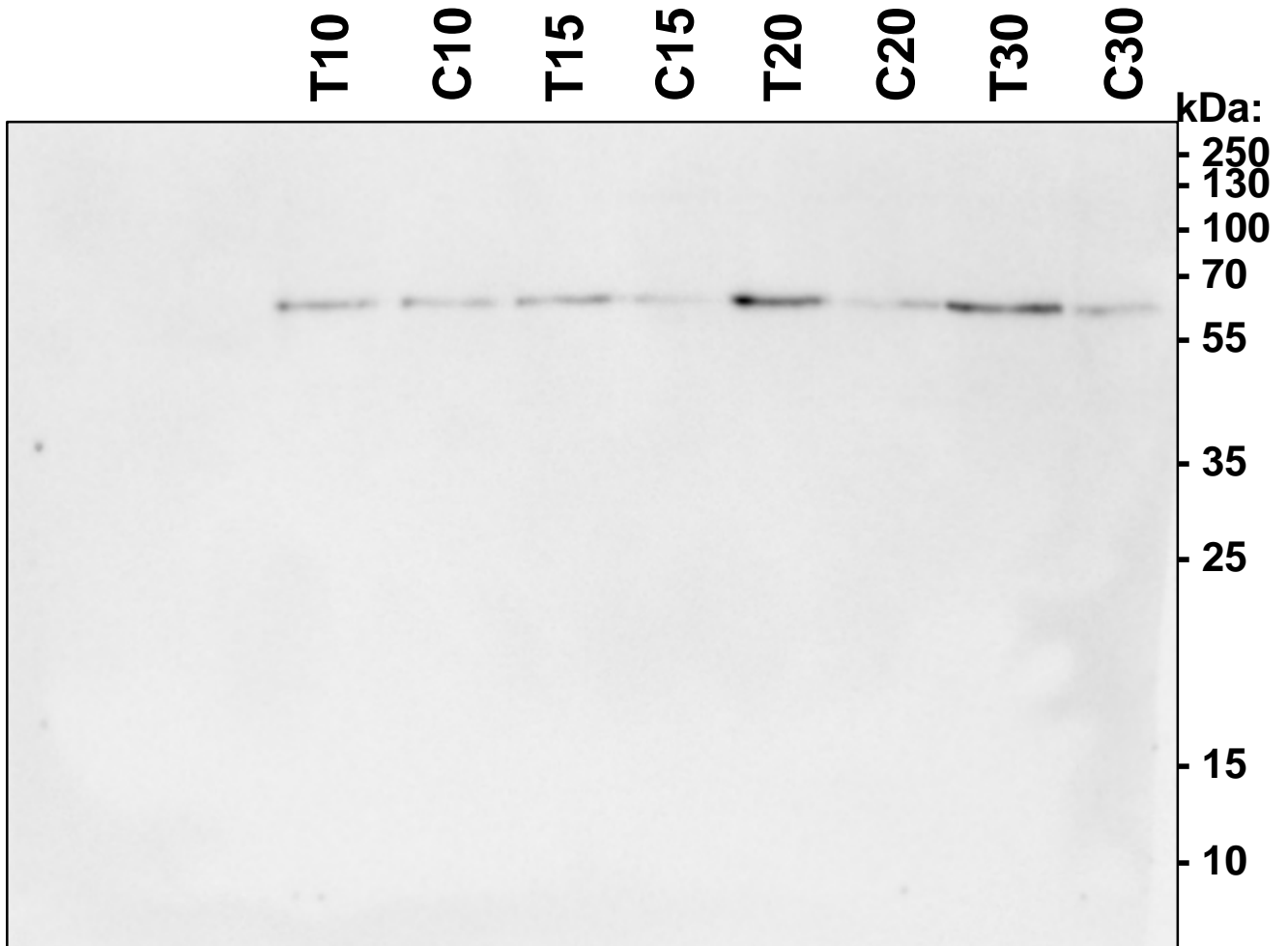

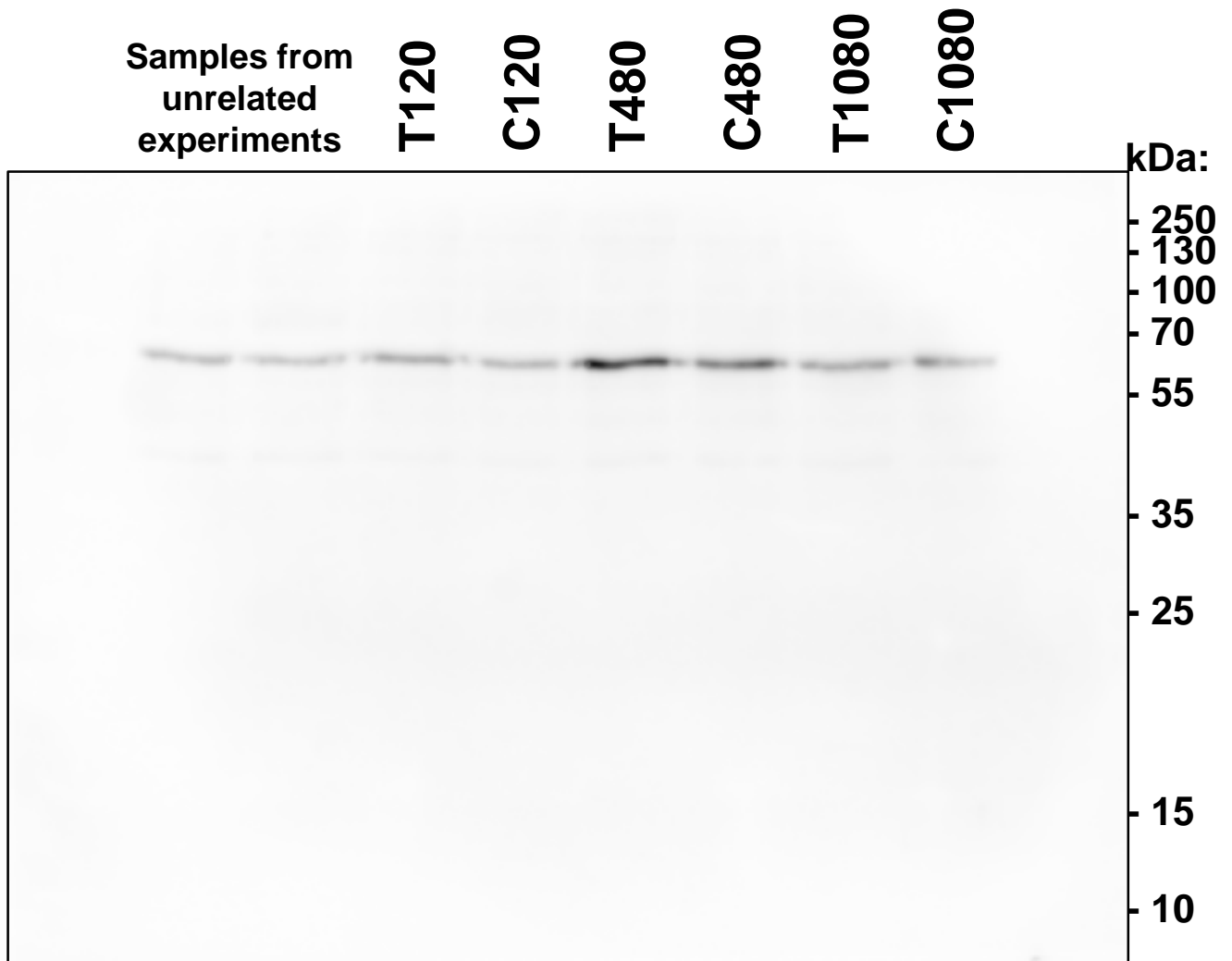

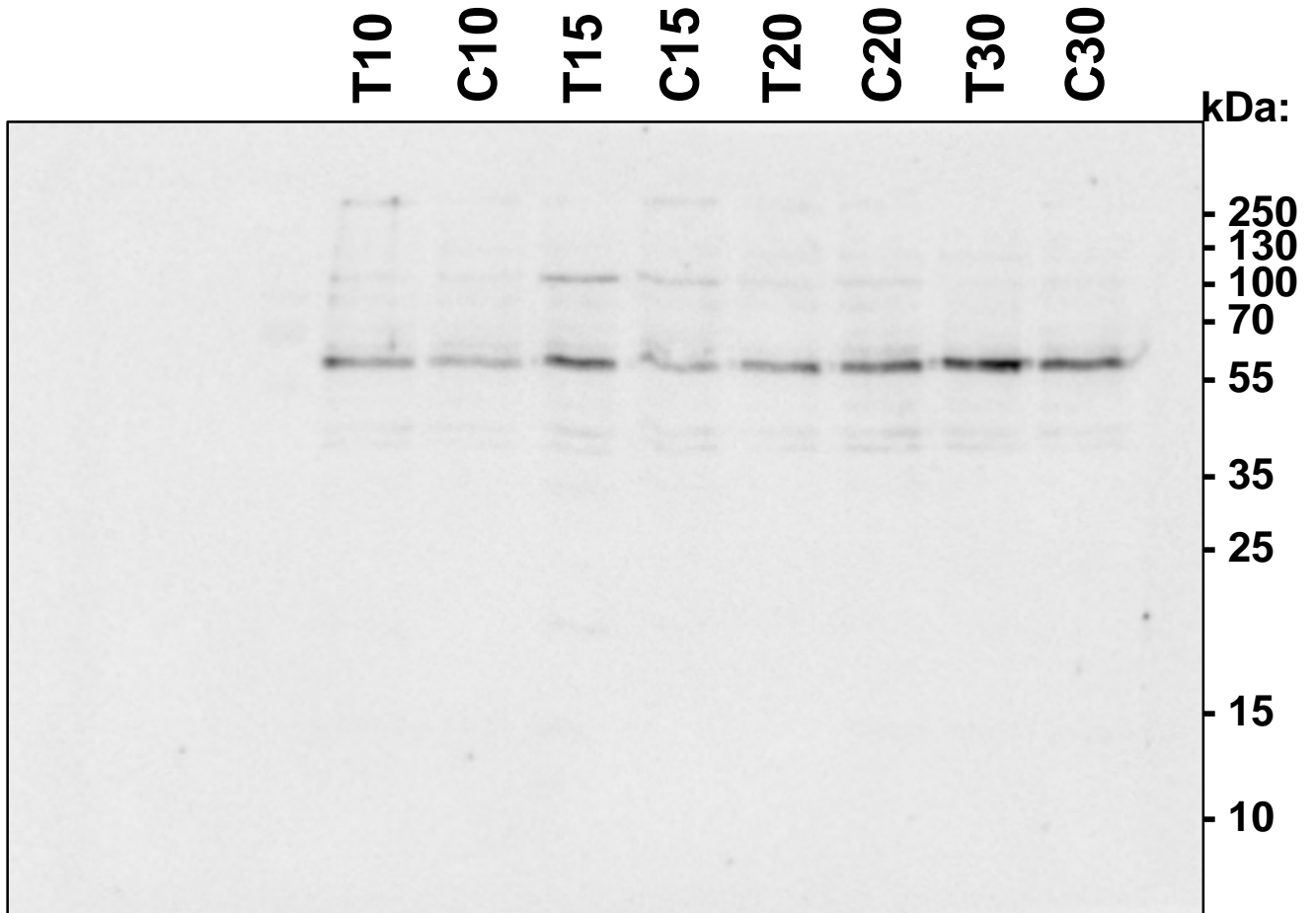

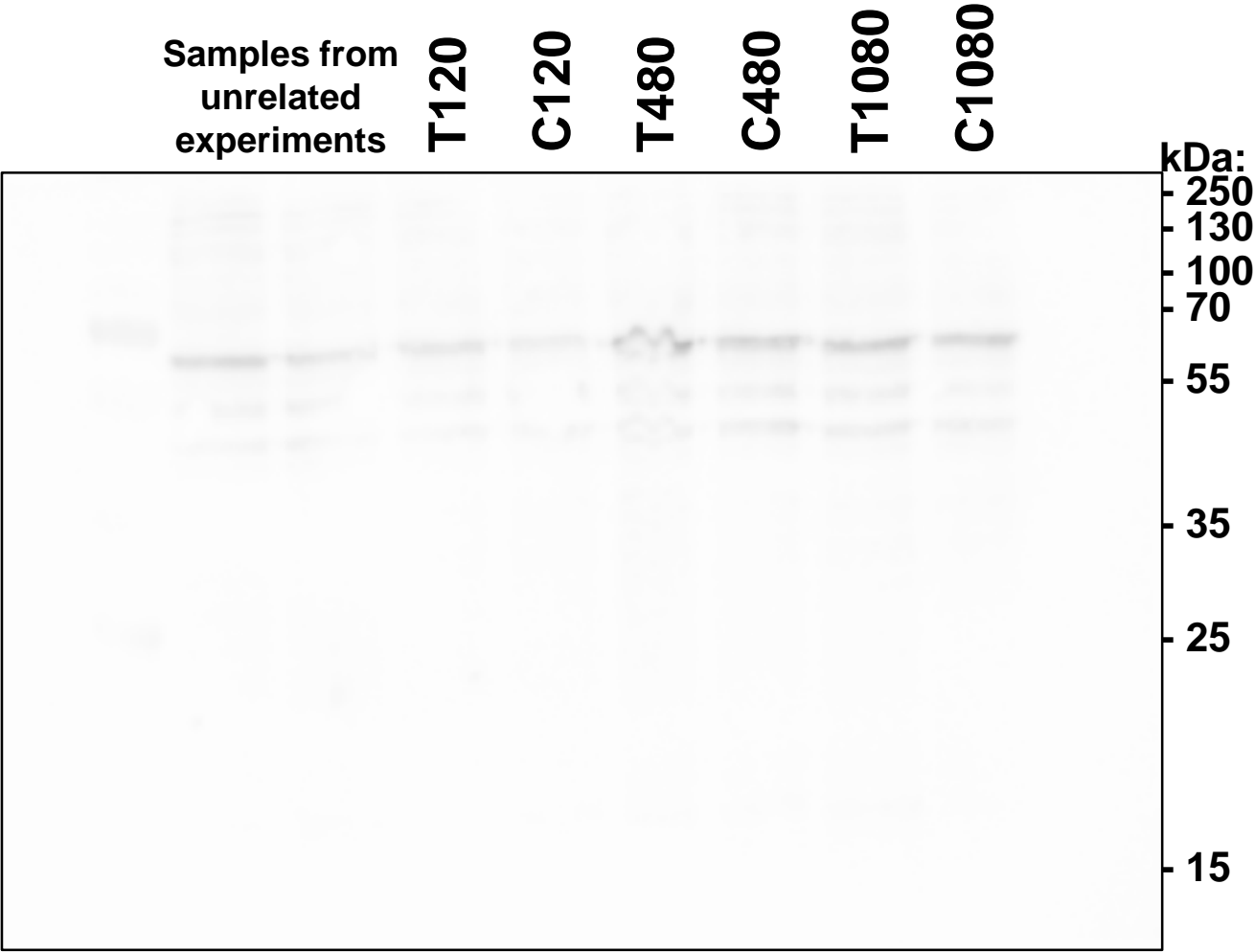

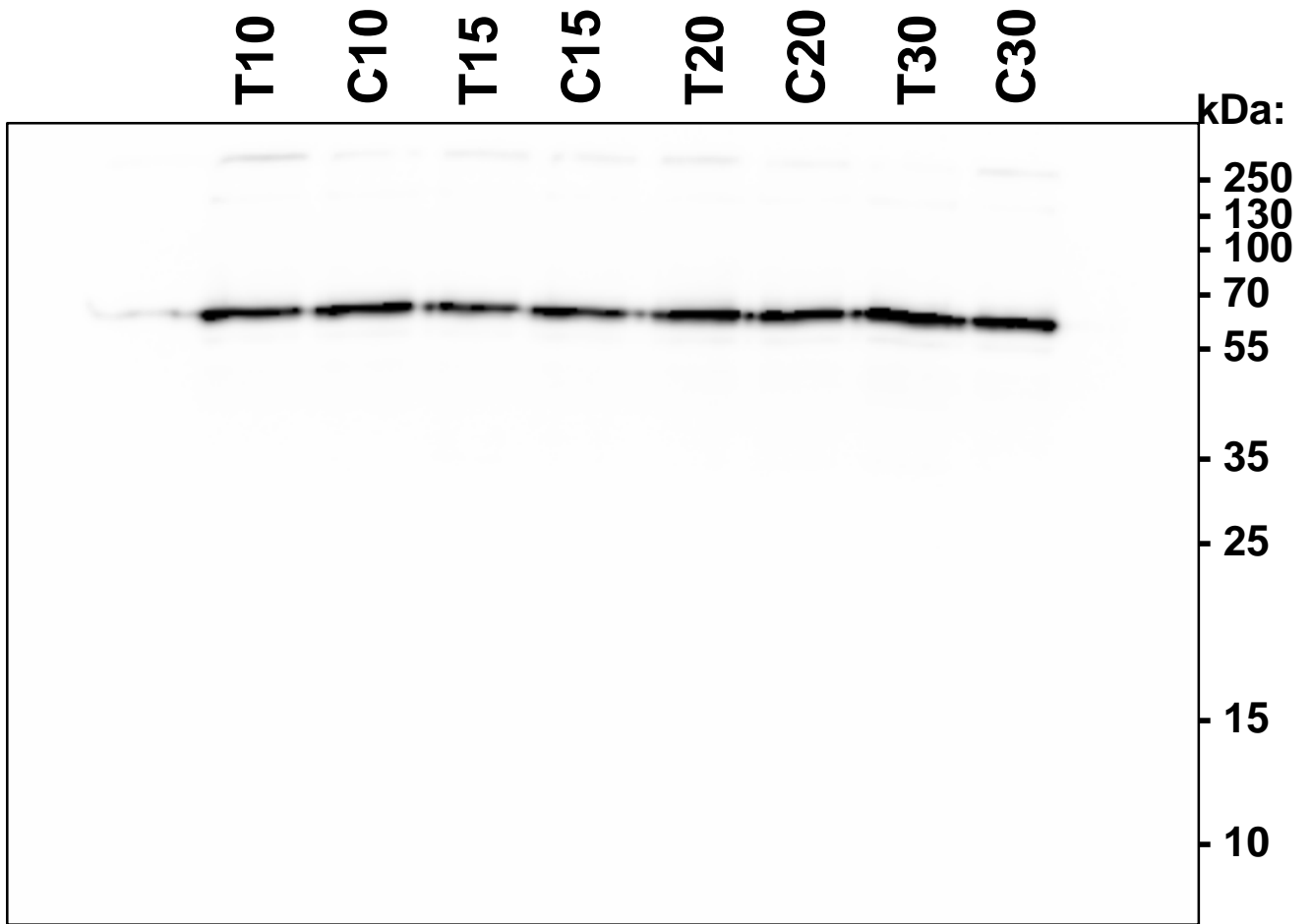

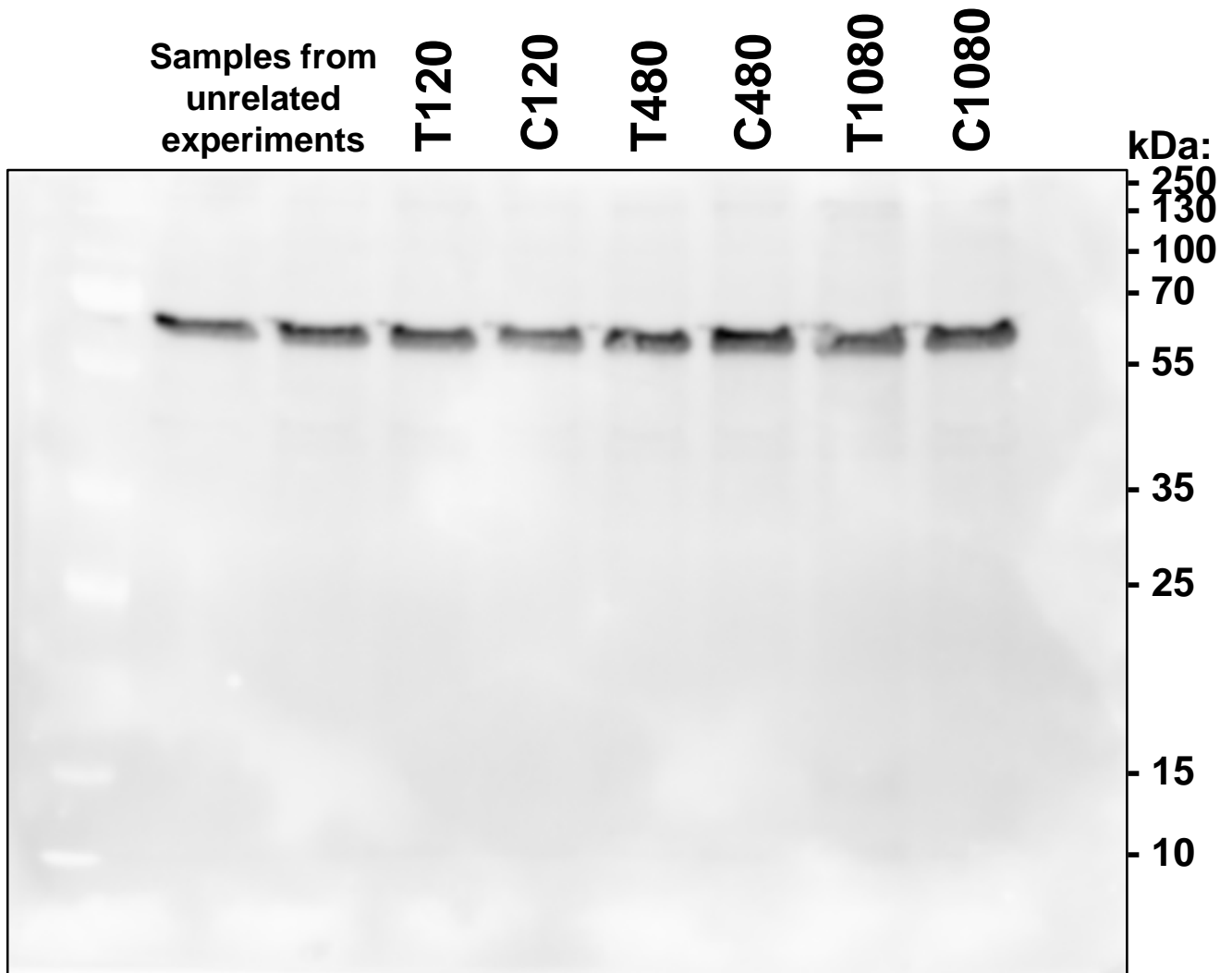

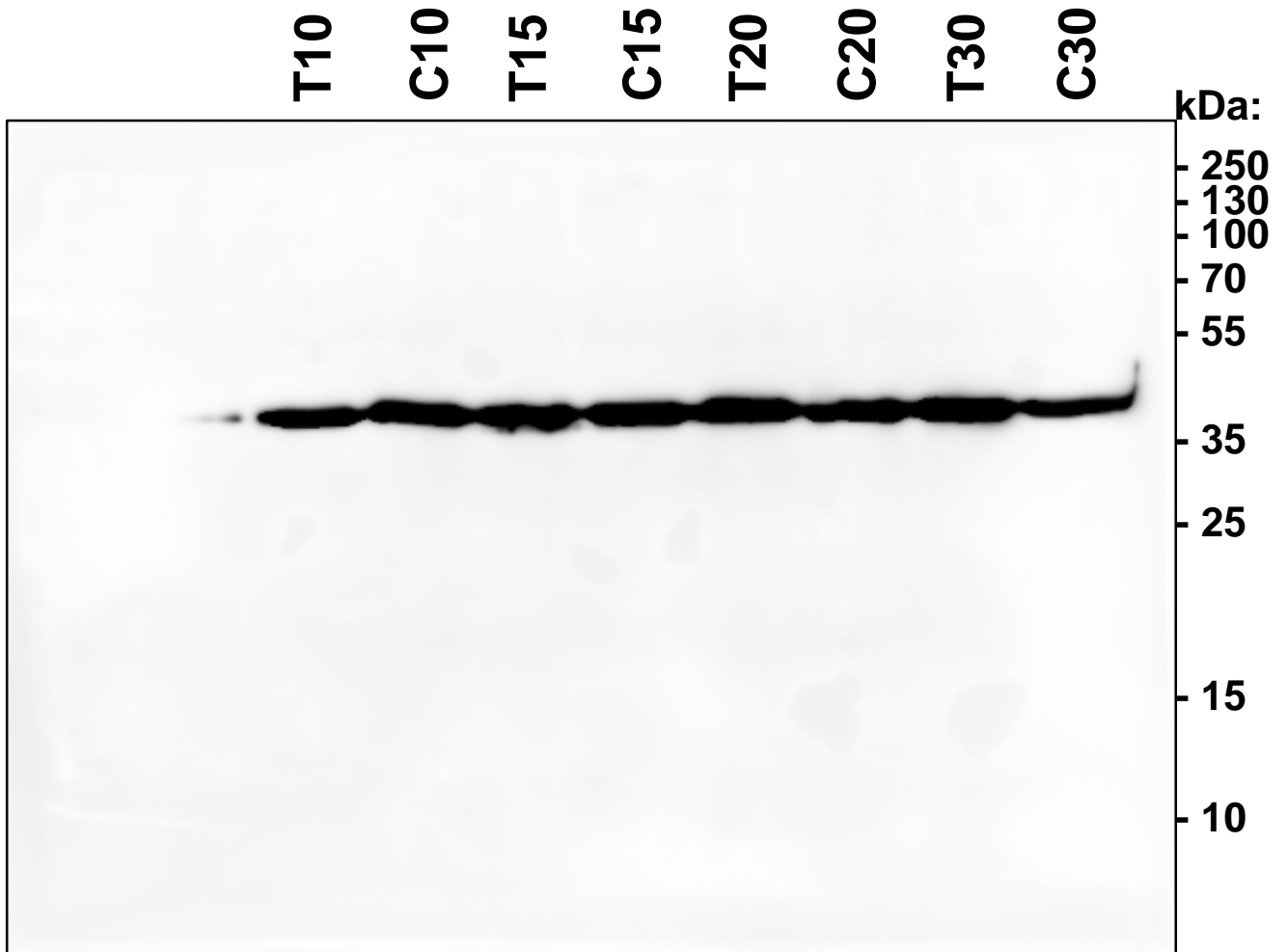

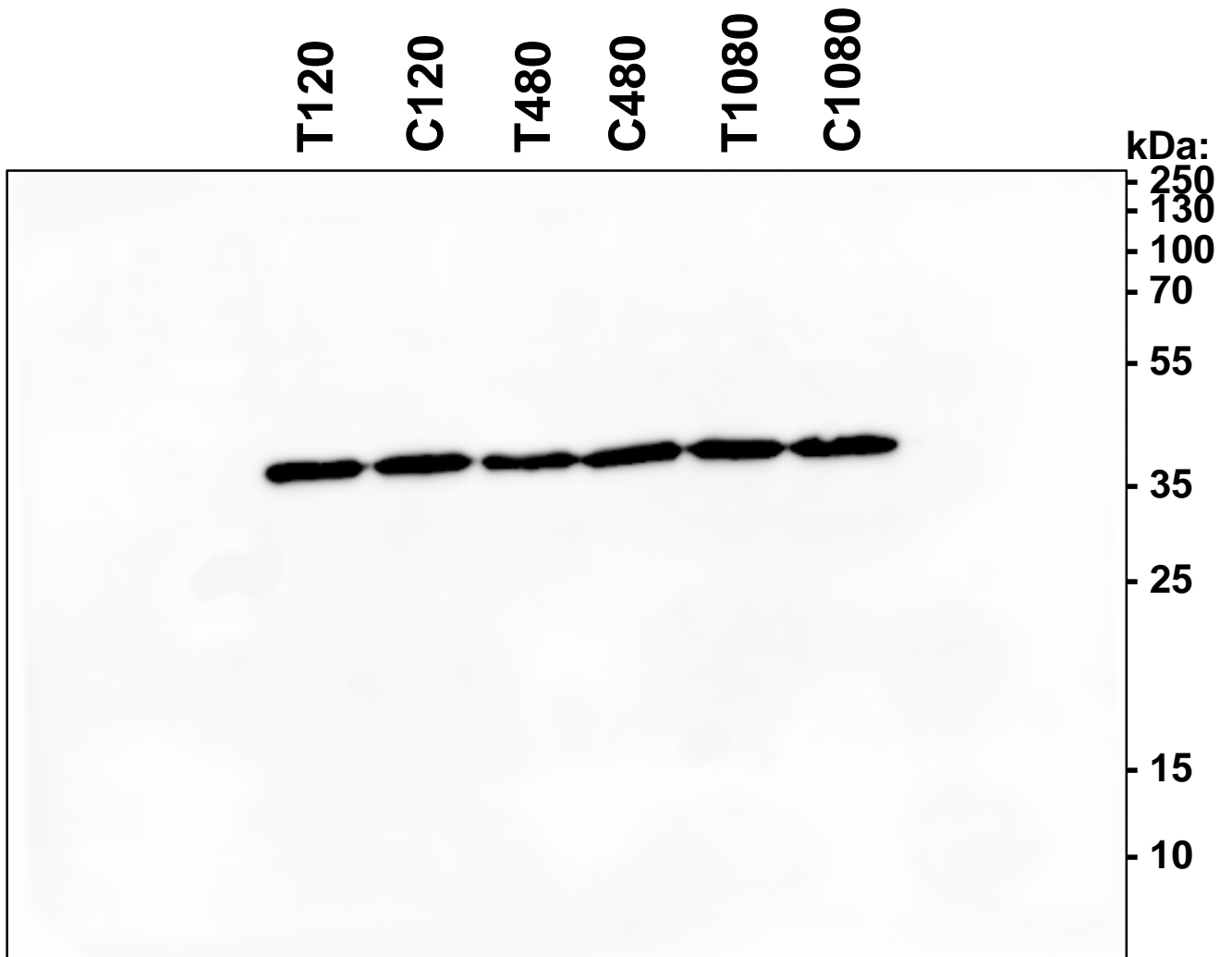

Supplement: S8 Fig — THP-1 macrophages were incubated for 0, 10, 15, 20, 30, 120, 420, or 1080 minutes with either a vehicle control (Control or C), or a 0.68 mM mixture of purified FFA (T) that matched the ratios observed post-hydrolysis of total human lipoprotein lipids by LPL. Cell lysates were collected and proteins were subjected to immunoblot analyses. Shown are one complete set of immunoblot results. A. pAkt (of Ser-473) at 10, 15, 20, and 30 minutes. B. pAkt (of Ser-473) at 120, 480, and 1080 minutes. C. pAkt (of Thr-308) at 10, 15, 20, and 30 minutes. D. pAkt (of Thr-308) at 120, 480, and 1080 minutes. E. Akt at 10, 15, 20, and 30 minutes. F. Akt at 120, 480, and 1080 minutes. G. β-actin at 10, 15, 20, and 30 minutes. H. β-actin at 120, 480, and 1080 minutes. (PDF) [file pone.0233180.s008.pdf]

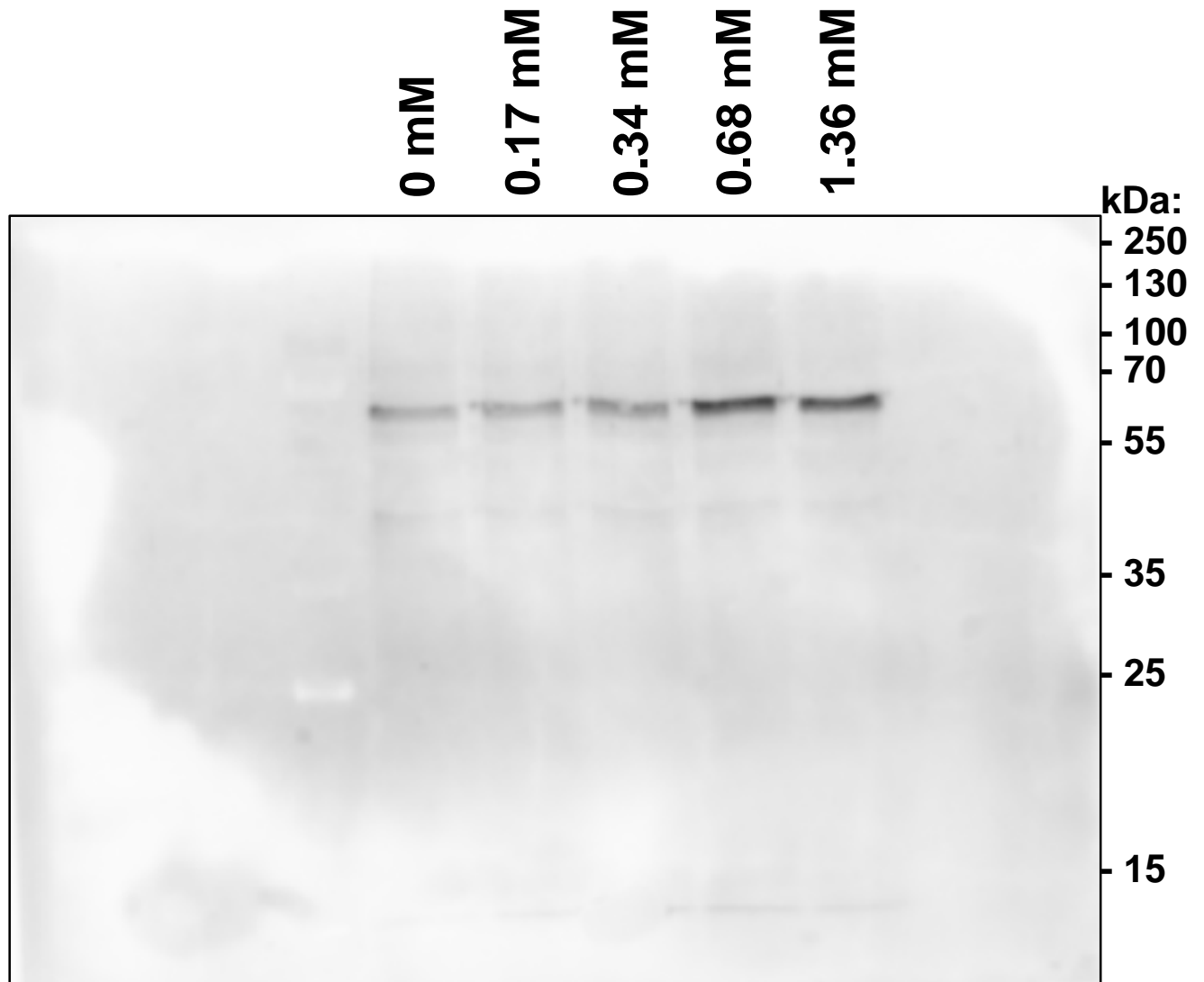

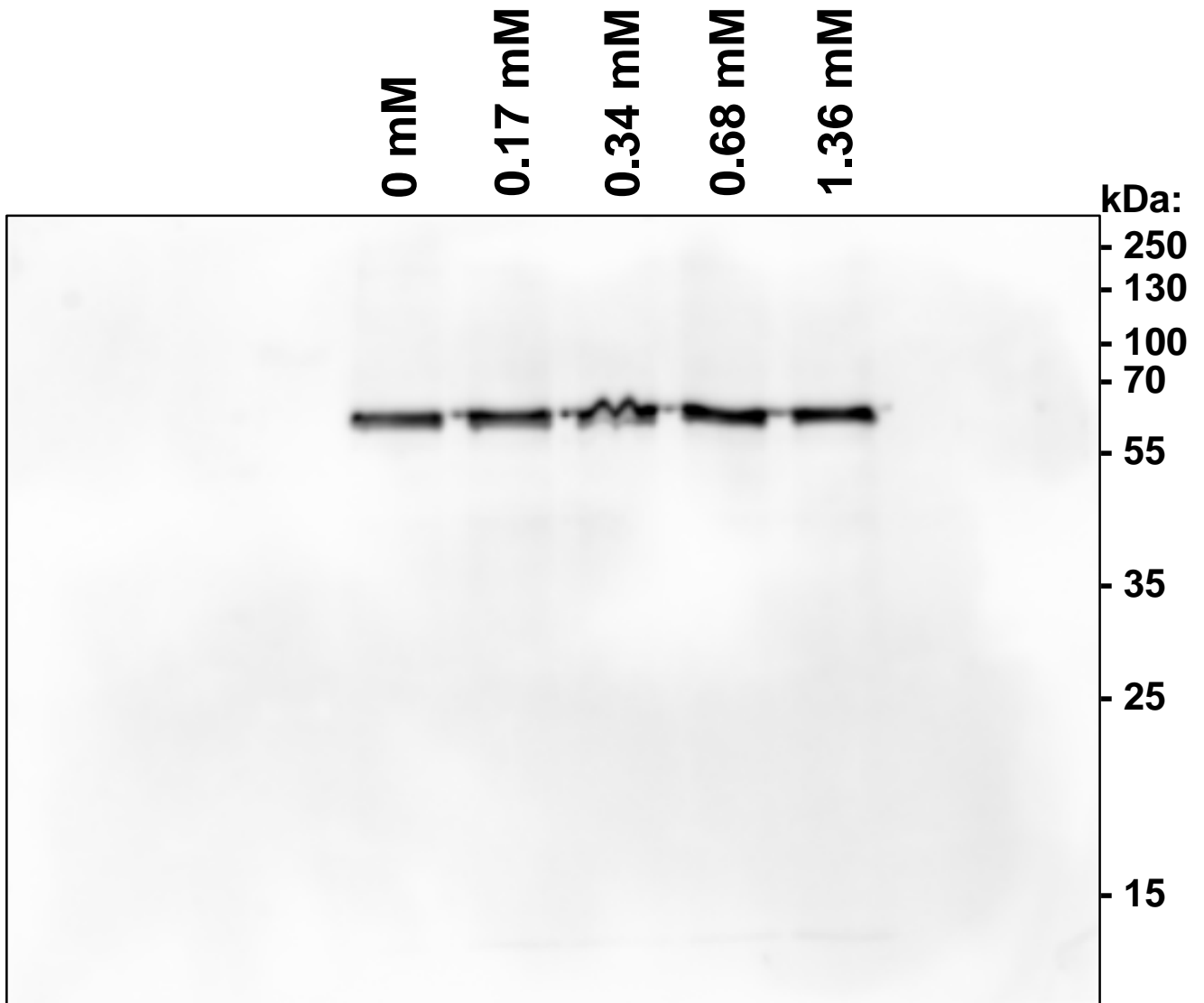

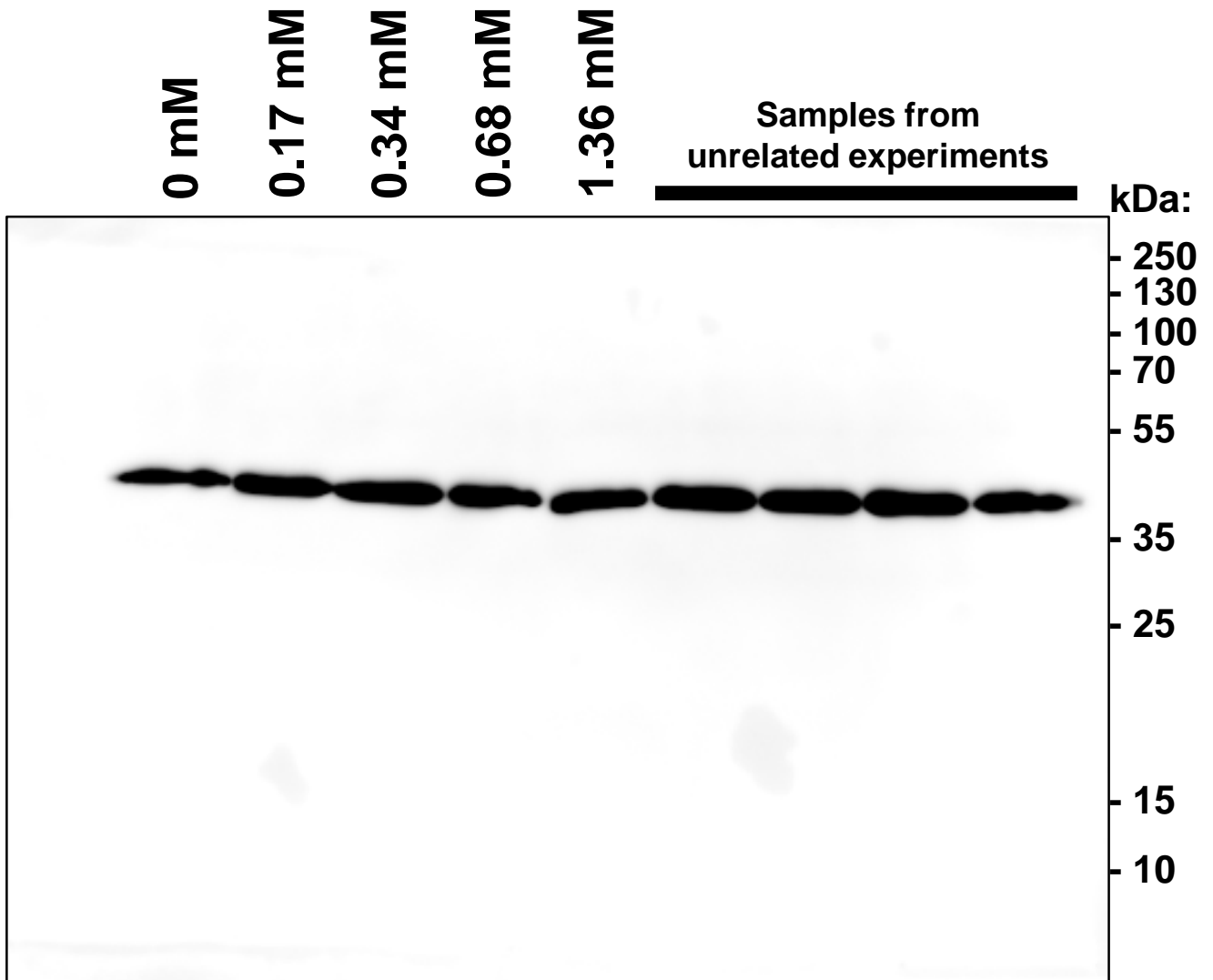

Supplement: S9 Fig — THP-1 macrophages were incubated for 2 hours with either a vehicle control (Control), or a mixture of purified FFA that matched the ratios observed post-hydrolysis of total human lipoprotein lipids by LPL; concentrations of 0 mM, 0.17 mM, 0.34 mM, 0.68 mM, and 1.38 mM were tested. Cell lysates were collected and proteins were subjected to immunoblot analyses. Shown are one complete set of immunoblot results. A. pAkt (of Ser-473). B. Akt. C. β-actin. (PDF) [file pone.0233180.s009.pdf]

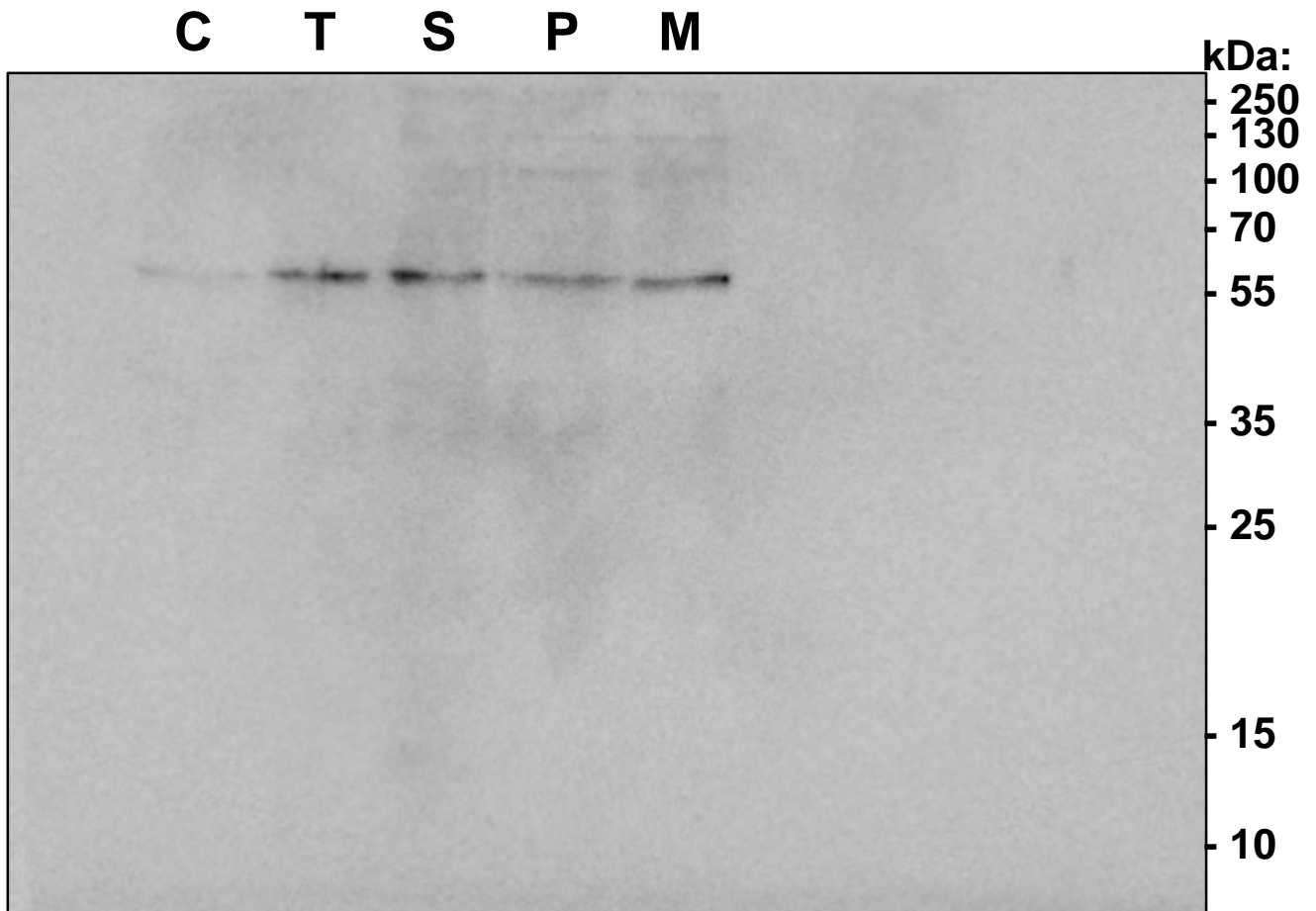

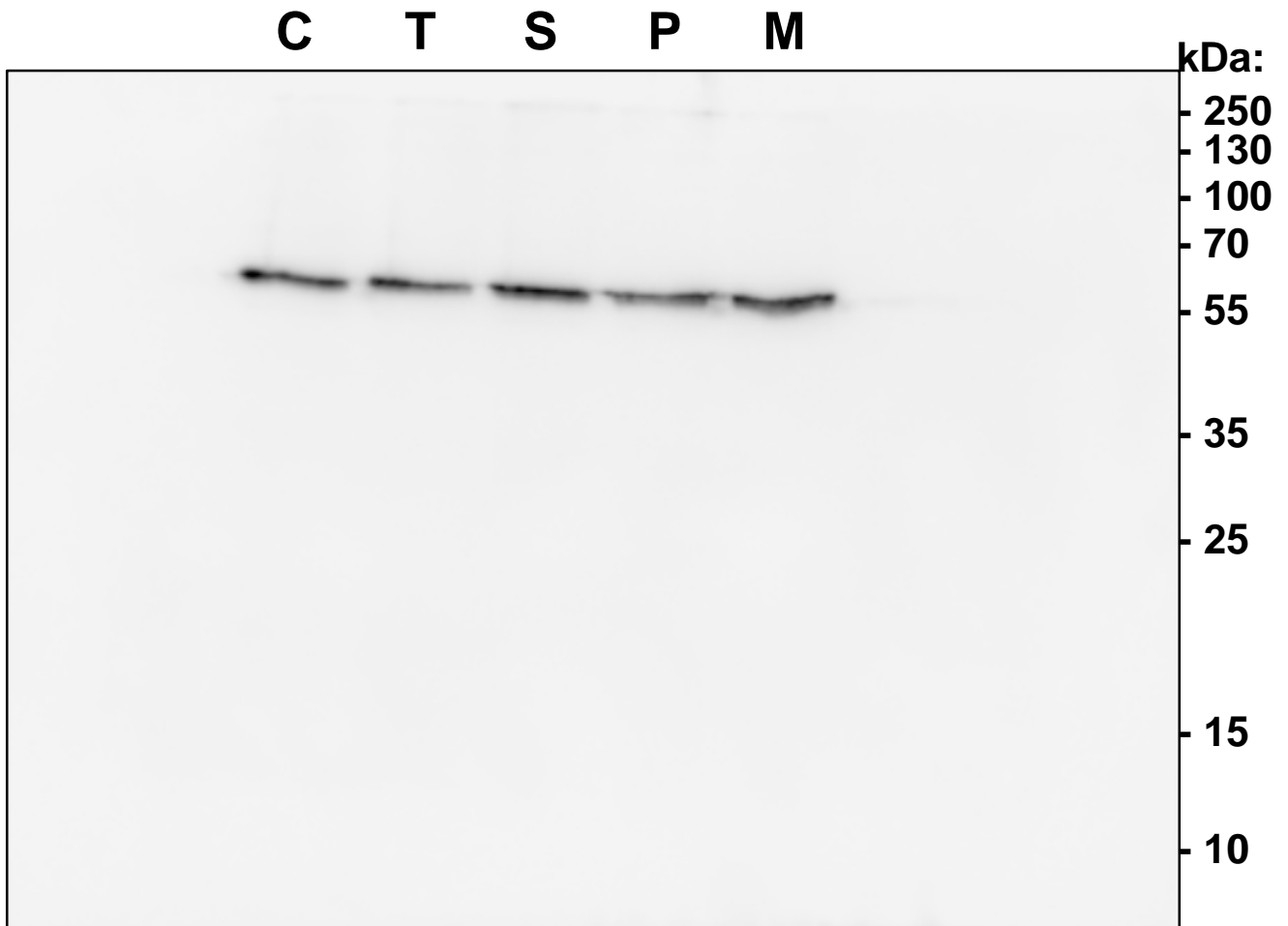

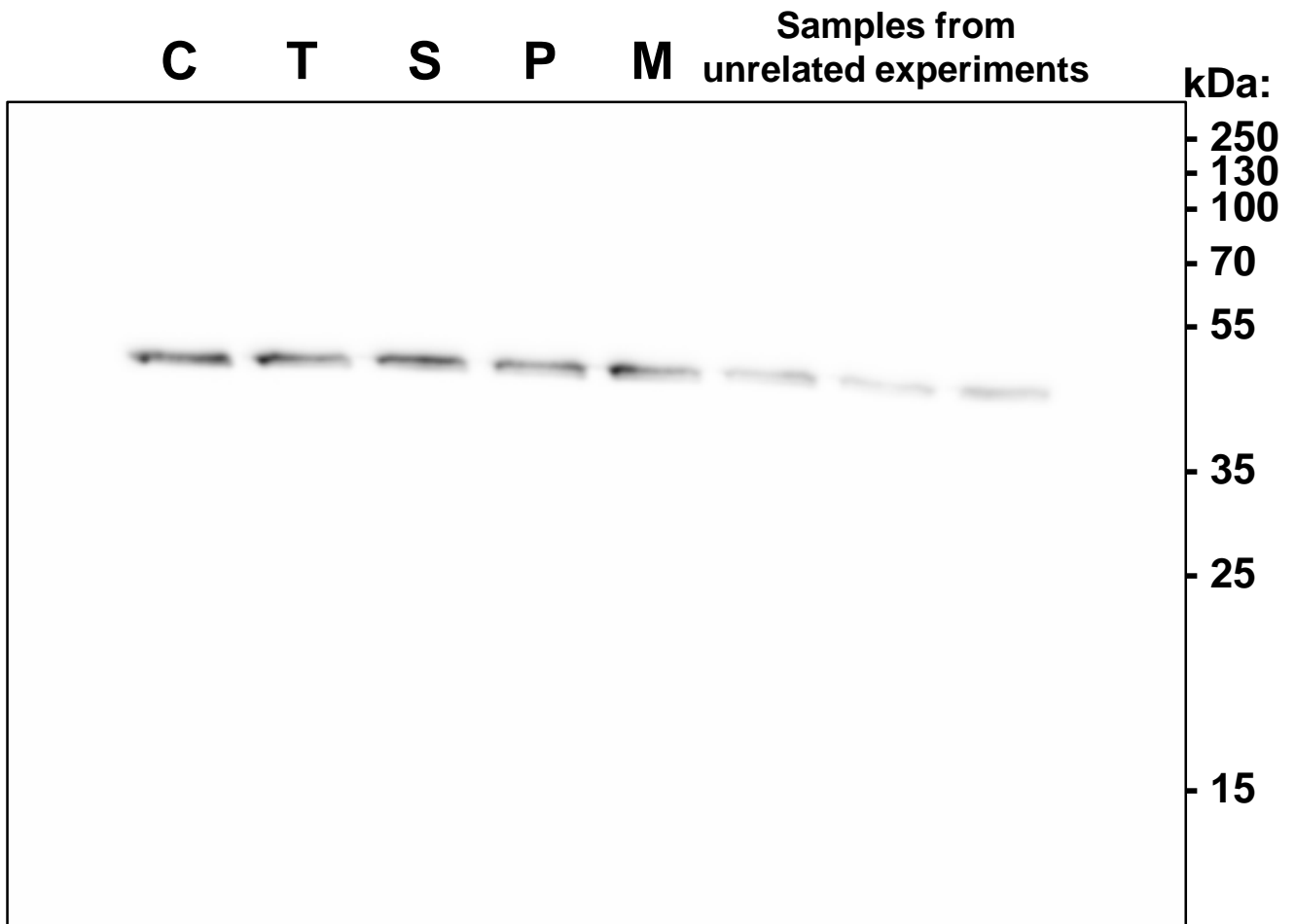

Supplement: S10 Fig — THP-1 macrophages were incubated for 2 hours with either a vehicle control (Control or C), a 0.68 mM mixture of purified FFA (Total or T) that matched the ratios observed post-hydrolysis of total human lipoprotein lipids by LPL, the SFA component of the total mixture (S), the MUFA component of the total mixture (M), or the PUFA component of the total mixture (P). Cell lysates were collected and proteins were subjected to immunoblot analyses. A. pAkt (of Ser-473). B. Akt. C. β-actin. (PDF) [file pone.0233180.s010.pdf]

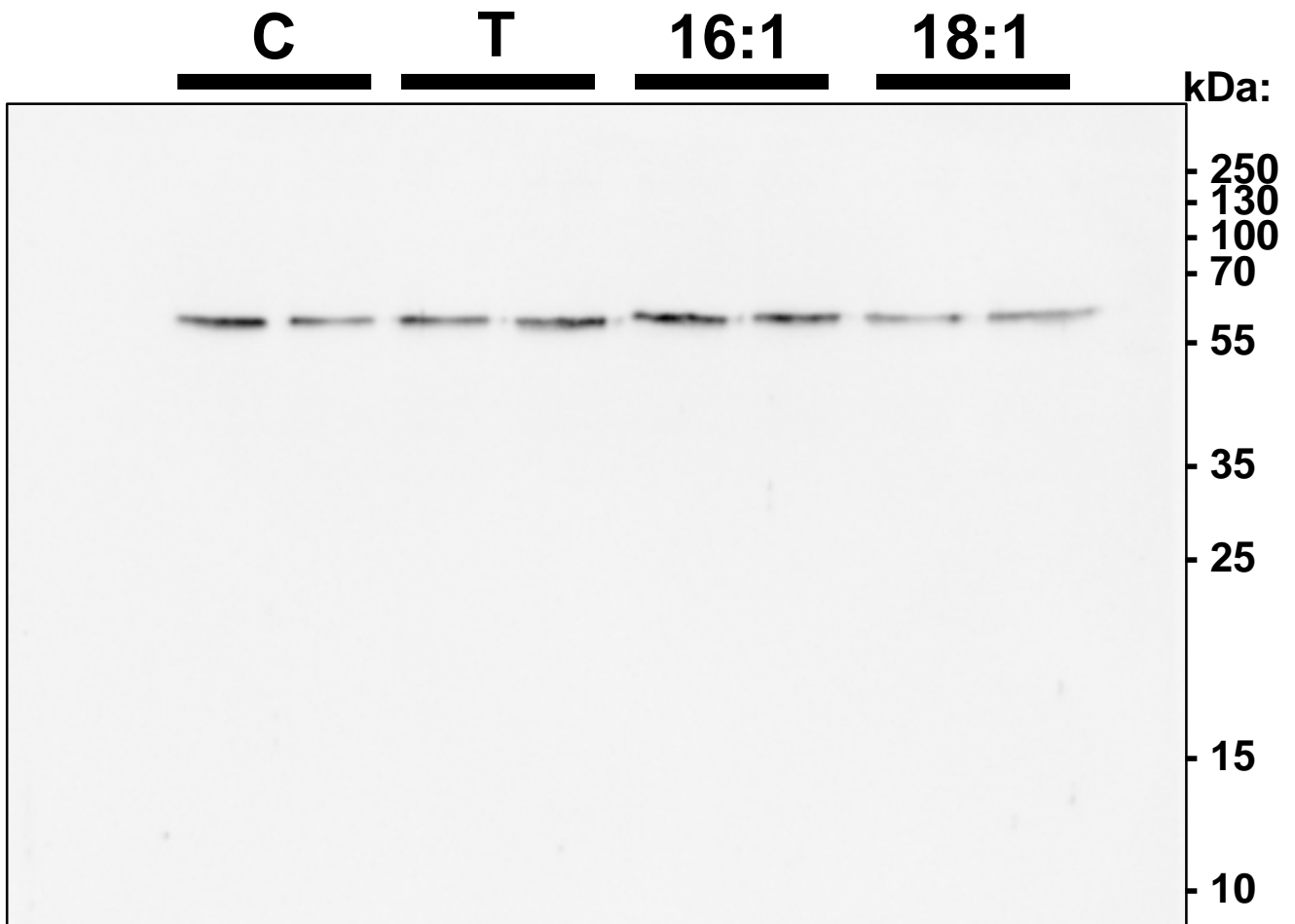

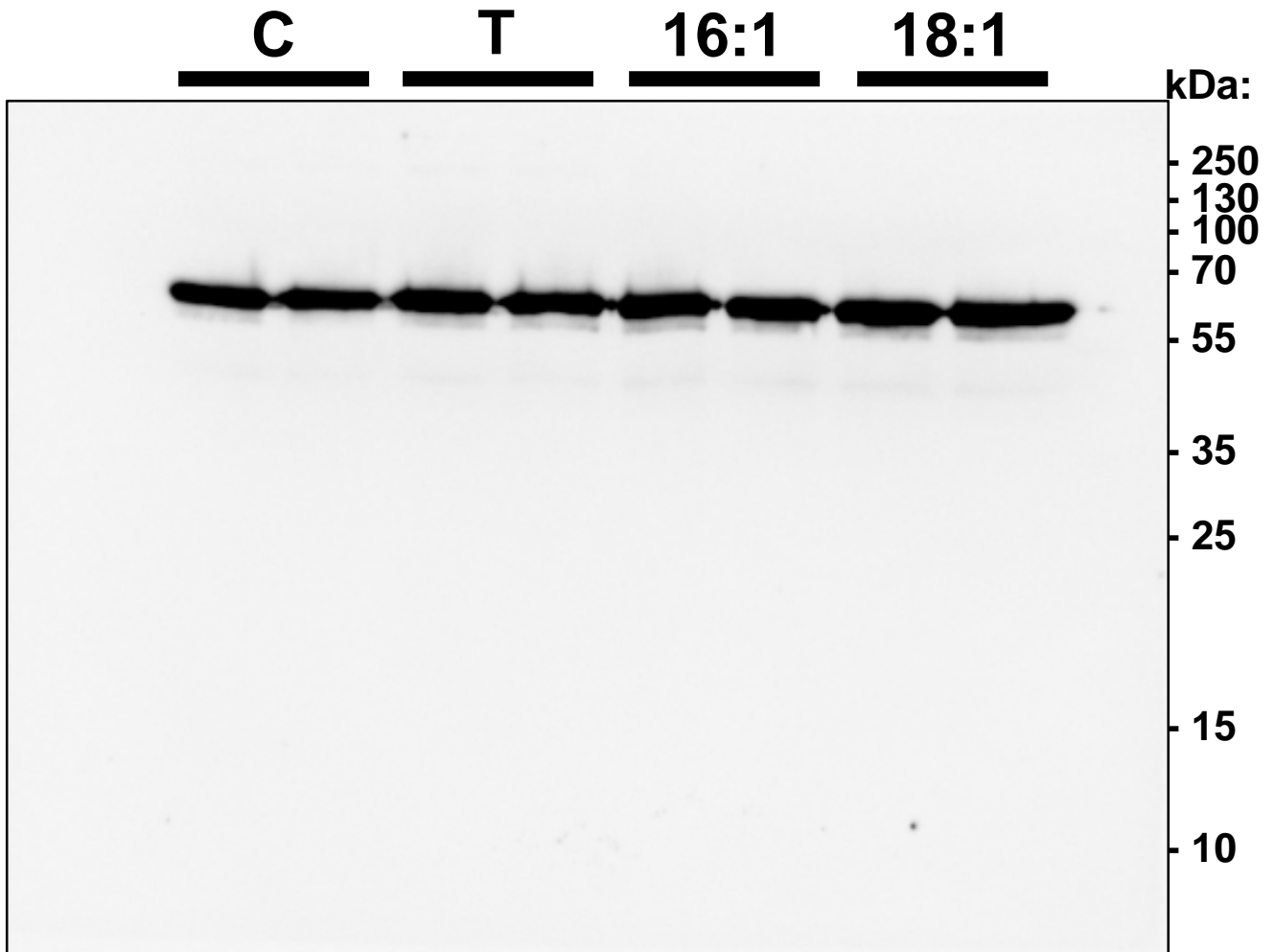

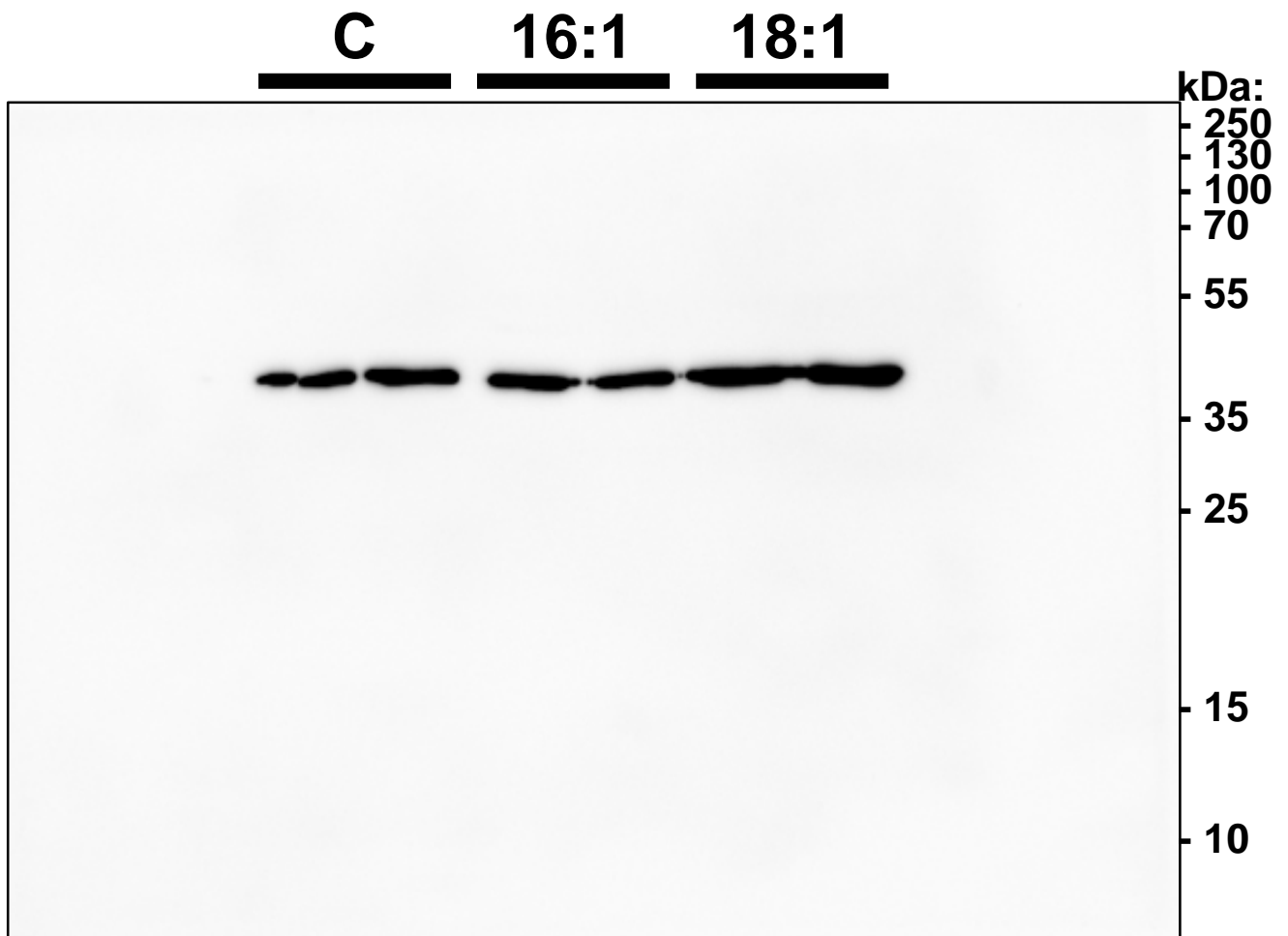

Supplement: S11 Fig — THP-1 macrophages were incubated for 2 hours with either a vehicle control (Control or C), 0.02 mM palmitoleate (16:1n-7 or 16:1), or 0.24 mM oleate (18:1n-9 or 18:1). Cell lysates were collected and proteins were subjected to immunoblot analyses. A. pAkt (of Ser-473). B. Akt. C. β-actin. Note, “A” and “B” also include the total FFA mixture at 0.68 mM but these data were not part of Fig 2B. (PDF) [file pone.0233180.s011.pdf]

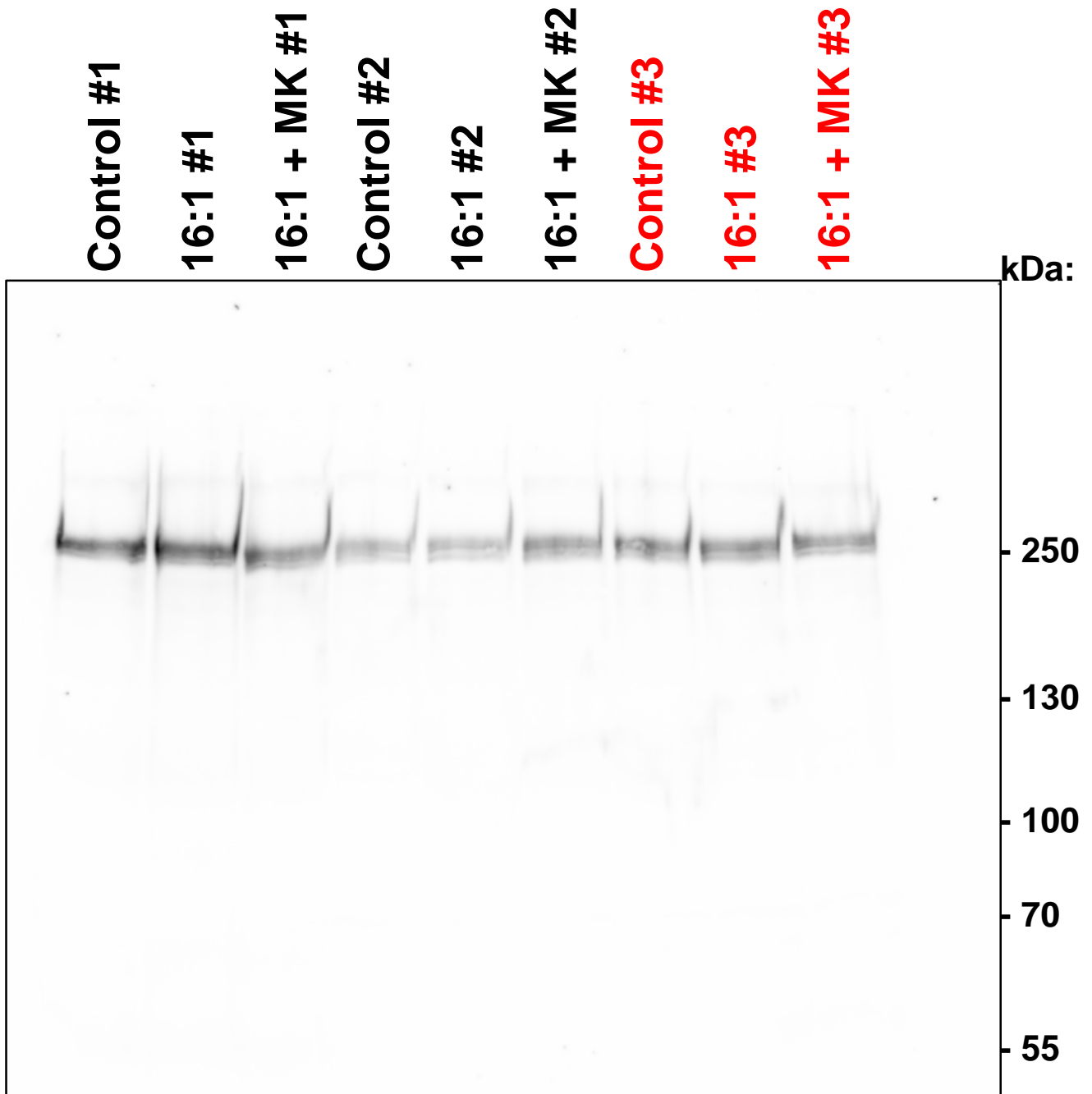

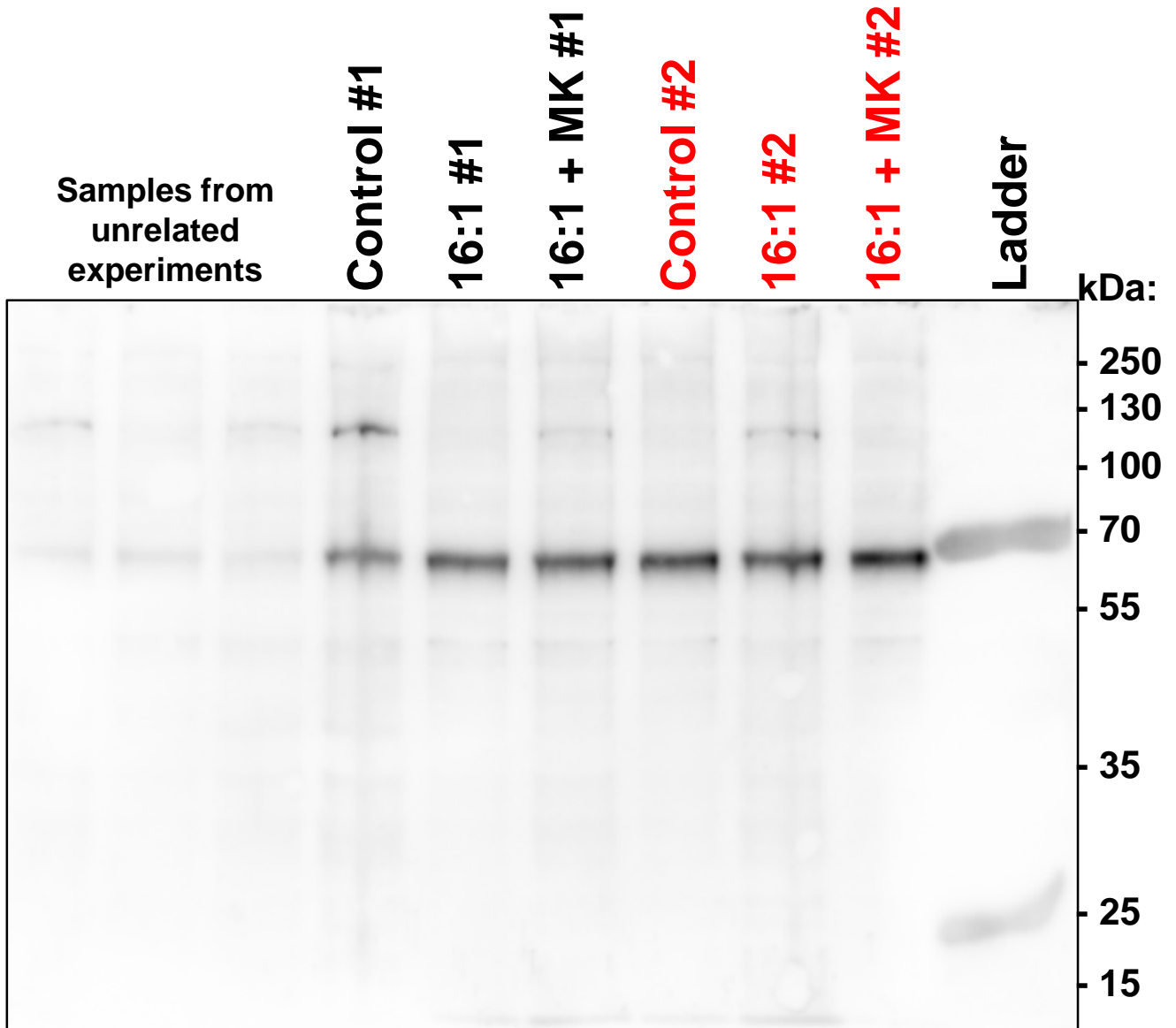

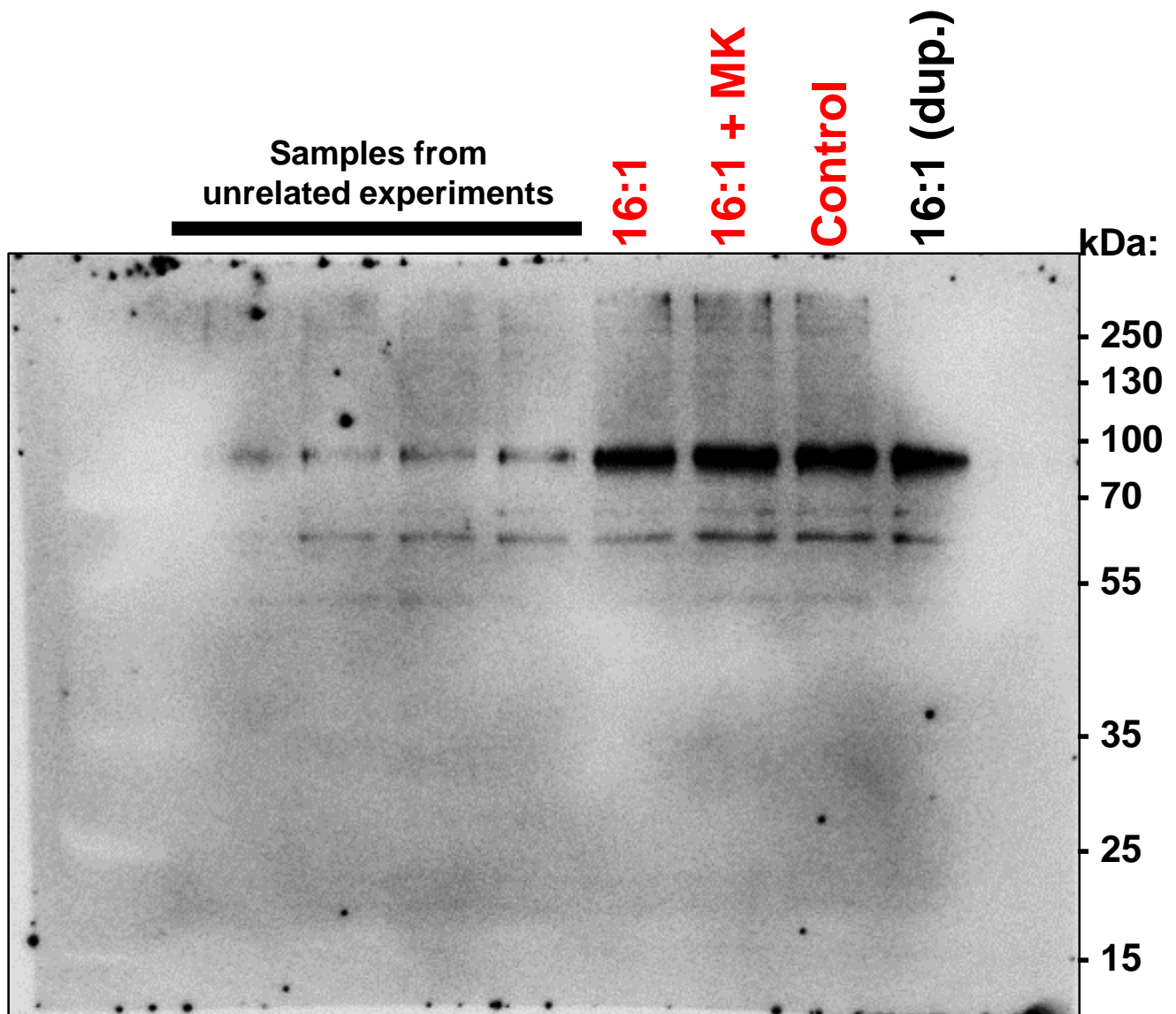

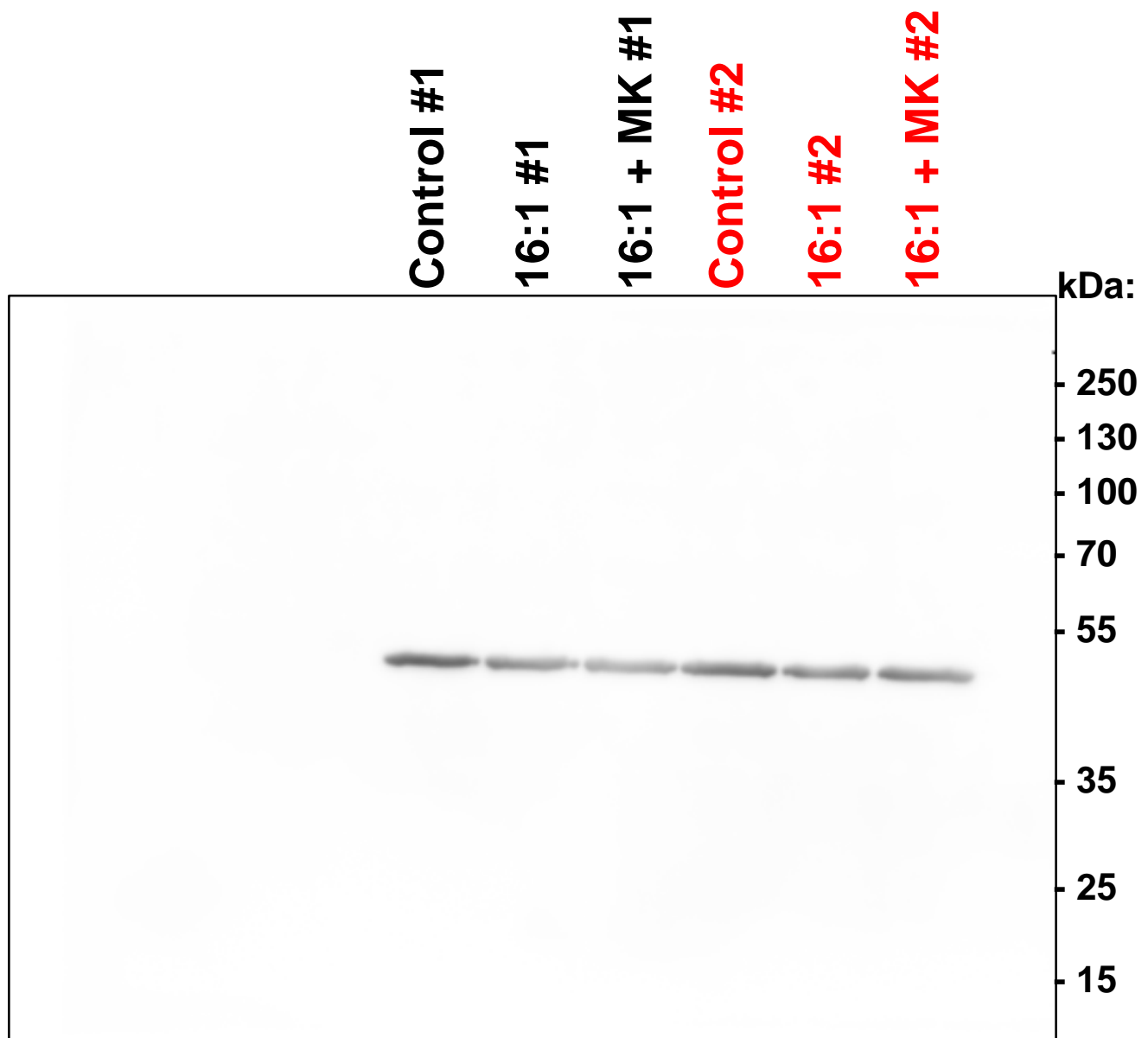

Supplement: S12 Fig — THP-1 macrophages were incubated for 18 hours with either a vehicle control (Control), 0.02 mM palmitoleate (16:1), or 0.02 mM palmitoleate in the presence of 1 μM MK-2206 (+ MK). Cell lysates were collected and proteins were subjected to immunoblot analyses. A. ABCA1. B. ABCG1. C. SR-BI. D. β-actin. Note, lanes labelled with red text were used for S4 Fig. (PDF) [file pone.0233180.s012.pdf]
