# Supplementary material for: THP-1 macrophage cholesterol efflux is impaired by palmitoleate through Akt activation
Source: PLoS One. 2020 May 21;15(5):e0233180. doi: 10.1371/journal.pone.0233180 (PMC7241781; doi:10.1371/journal.pone.0233180)
Supplement: S1 File — Materials and methods associated with S3, S6 and S7 Figs. (PDF) [file pone.0233180.s013.pdf]

Supporting materials and methods

Cholesterol efflux (for S3 Fig data)

ApoA-I (Lee Biosolutions, Maryland Heights, MO, USA) was salt-exchanged using a PD-10 desalting column (GE Healthcare) equilibrated with phosphate-buffered saline, and the protein concentration was measured using the absorbance at 280 nm and the molar absorption coefficient of 1.23 ml/ mg·cm [1]. Cholesterol efflux assays using THP-1 macrophages that were not treated with acetylated LDL were carried out as previously described [2]. In brief, cells were cultured in 12-well plates and radiolabelled with 1 μCi/ml [^{3}H]cholesterol (PerkinElmer, Waltham, MA, USA). Following labelling, cells were incubated for 18 hours with media containing 23.7 μM palmitoleate or vehicle control. After incubation without or with fatty acid, cells were incubated without or with 50 μg/ml apoA-I, or 50 μg/ml HDL (Lee Biosolutions) for 6 hours. After 6 hours, media were collected and cells were lysed using 1 ml of 0.2 M NaOH for 30 minutes; [^{3}H]cholesterol from media and cell lysates were quantified by scintillation counting. The amount of [^{3}H]cholesterol effluxed was calculated as a percentage of [^{3}H]cholesterol effluxed into the medium per amount of total cell and medium [^{3}H]cholesterol. Background efflux (in the absence of apoA-I) was subtracted from efflux data to obtain apoA-I specific efflux. For cholesterol loading using acetylated LDL, during radiolabelling, cells were also incubated with 50 μg/ml acetylated LDL (Lee Biosolutions).

Oil Red O Staining

As described under Materials and Methods, THP-1 macrophages were incubated for 18 hours with the total FFA mixture (with FFA concentrations matching those within the lipoprotein hydrolysis products generated by LPL), in the absence or presence of 100 μM of the PI3K inhibitor LY294002. Following incubation, the cells were washed twice with phosphate-buffered saline (PBS, pH 7.4) and stained with 0.36% *w/v* Oil red O in 60% v/v isopropanol (Millipore, Toronto, ON Canada) for 15 minutes at room temperature. After 15 minutes, cells were washed three times with PBS and then incubated for 30 minutes with 1 ml isopropanol. The isopropanol (containing extracted stain) was removed and the absorbance at 520 nm was measured.

Expression of ABCA1, ABCG1, and SR-BI mRNA

As described under Materials and Methods, THP-1 macrophages were incubated for 18 hours with the total FFA mixture (with FFA concentrations matching those within the lipoprotein hydrolysis products generated by LPL), in the absence or presence of 1 μM of MK-2206. RNA was isolated and real-time PCR was carried out using previously reported primers and methods to determine *ABCA1*, *ABCG1*, *SCARB1*, and *ACTB* expression [2]. All data were corrected for primer pair efficiencies.

Supplementary References

1. Gillotte KL, Zaiou M, Lund-Katz S, Anantharamaiah GM, Holvoet P, Dhoest A, et al. Apolipoprotein-mediated plasma membrane microsolubilization: role of lipid affinity and

membrane penetration in the efflux of cellular cholesterol and phospholipid. J Biol Chem. 1999;274(4):2021-2028. doi: 10.1074/jbc.274.4.2021

2. Yang Y, Thyagarajan N, Coady BM, Brown RJ. Cholesterol efflux from THP-1 macrophages is impaired by the fatty acid component from lipoprotein hydrolysis by lipoprotein lipase. Biochem Biophys Res Commun 2014;451(4):632-636. doi: 10.1016/j.bbrc.2014.08.040
